# Publisher Correction: Enhancer remodeling promotes tumor-initiating activity in NRF2-activated non-small cell lung cancers

**DOI:** 10.1038/s41467-021-20927-9

**Published:** 2021-01-15

**Authors:** Keito Okazaki, Hayato Anzawa, Zun Liu, Nao Ota, Hiroshi Kitamura, Yoshiaki Onodera, Md. Morshedul Alam, Daisuke Matsumaru, Takuma Suzuki, Fumiki Katsuoka, Shu Tadaka, Ikuko Motoike, Mika Watanabe, Kazuki Hayasaka, Akira Sakurada, Yoshinori Okada, Masayuki Yamamoto, Takashi Suzuki, Kengo Kinoshita, Hiroki Sekine, Hozumi Motohashi

**Affiliations:** 1 Department of Gene Expression Regulation, Institute of Development, Aging and Cancer, Tohoku University, Sendai, 980-8575 Japan; 2 Department of System Bioinformatics, Graduate School of Information Sciences, Tohoku University, 980-8579 Sendai, Japan; 3 Department of Anatomic Pathology, Tohoku University Graduate School of Medicine, Sendai, 980-8575 Japan; 4 Department of Integrative Genomics, Tohoku Medical Megabank Organization, Tohoku University, Sendai, 980-8573 Japan; 5 Department of Pathology, Tohoku University Hospital, Sendai, 980-8575 Japan; 6 Department of Thoracic Surgery, Institute of Development, Aging and Cancer, Tohoku University, Sendai, 980-8575 Japan; 7 Department of Medical Biochemistry, Tohoku University Graduate School of Medicine, Sendai, 980-8575 Japan; 8 Department of Pathology and Histotechnology, Tohoku University Graduate School of Medicine, Sendai, 980-8575 Japan

**Keywords:** Non-small-cell lung cancer, Transcriptional regulatory elements

Correction to: *Nature Communications* 10.1038/s41467-020-19593-0, published online 20 November 2020.

This Article contained an error in Fig. 8. In Fig. 8h multiple white and blue bars denoting CEBPB were present in the histogram.

This has now been corrected in the PDF and HTML versions of the Article.

