# Supplementary material for: Enhancer remodeling promotes tumor-initiating activity in NRF2-activated non-small cell lung cancers
Source: Nat Commun. 2020 Nov 20;11:5911. doi: 10.1038/s41467-020-19593-0 (PMC7679411; doi:10.1038/s41467-020-19593-0)
Supplement: Supplementary file 1 — Supplementary Information [file 41467_2020_19593_MOESM1_ESM.pdf]

Supplementary Information

Enhancer Remodeling Promotes Tumor-Initiating Activity in NRF2-Activated Non-Small Cell Lung Cancers

Keito Okazaki[1], Hayato Anzawa[2], Zun Liu[1], Nao Ota[1], Hiroshi Kitamura[1], Yoshiaki Onodera[3], Md. Morshedul Alam[1], Daisuke Matsumaru[1], Takuma Suzuki[1], Fumiki Katsuoka[4], Shu Tadaka[4], Ikuko Motoike[4], Mika Watanabe[5], Kazuki Hayasaka[1,6], Akira Sakurada[6], Yoshinori Okada[6], Masayuki Yamamoto[4,7], Takashi Suzuki[8], Kengo Kinoshita[2,4], Hiroki Sekine[1]*, Hozumi Motohashi[1]*

[1]Department of Gene Expression Regulation and [6]Department of Thoracic Surgery, Institute of Development, Aging and Cancer, Tohoku University, Sendai 980-8575, Japan
[2]Department of System Bioinformatics, Graduate School of Information Sciences, Tohoku University Sendai 980-8579, Japan
[3]Department of Anatomic Pathology, [7]Department of Medical Biochemistry, and [8]Department of Pathology and Histotechnology, Tohoku University Graduate School of Medicine, Sendai 980-8575, Japan
[4]Department of Integrative Genomics, Tohoku Medical Megabank Organization, Tohoku University, Sendai 980-8573, Japan
[5]Department of Pathology, Tohoku University Hospital, Sendai 980-8575, Japan

Short title: NRF2-NOTCH3 axis promotes tumor-initiating activity of cancer

***Corresponding authors**
Hiroki Sekine, Ph.D.,
Department of Gene Expression Regulation, Institute of Development, Aging and Cancer, Tohoku University.
4-1 Seiryo-cho, Aoba-ku, Sendai, Miyagi, 980-8575, Japan.
Phone: +81-22-717-8553
Fax: +81-22-717-8554
E-mail: sekine@med.tohoku.ac.jp.

Hozumi Motohashi, M.D., Ph.D.,
Department of Gene Expression Regulation, Institute of Development, Aging and Cancer, Tohoku University.
4-1 Seiryo-cho, Aoba-ku, Sendai, Miyagi, 980-8575, Japan.
Phone: +81-22-717-8550
Fax: +81-22-717-8554
E-mail: hozumim@med.tohoku.ac.jp.

Supplementary Fig. 1

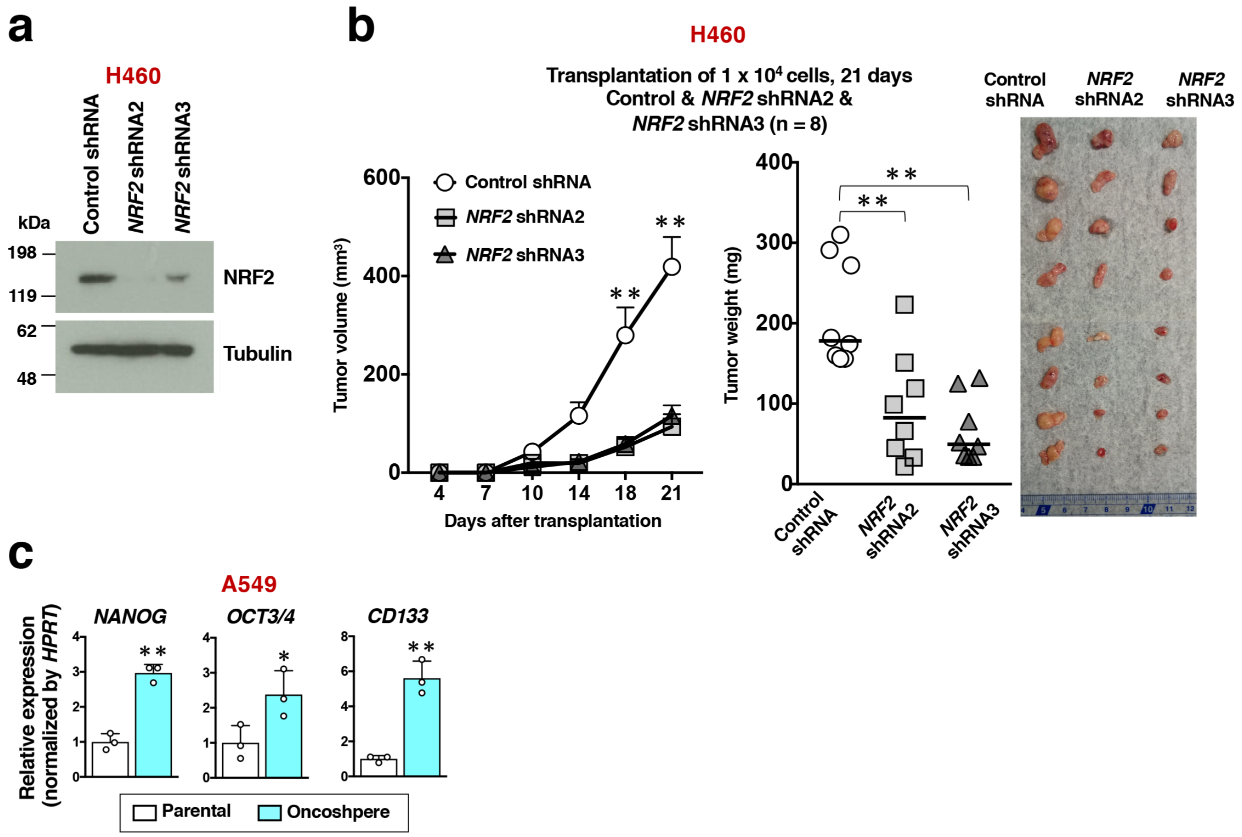

Supplementary Figure 1. Evaluation of the tumor-initiating activity of NRF2-activated NSCLCs.

a. Immunoblot analysis of NRF2 protein levels in inducible *NRF2*-knockdown H460 cells (*NRF2* shRNA2 and *NRF2* shRNA3) and control H460 cells after 48 hrs of tetracycline treatment. Two different shRNAs were used for inducible knockdown of *NRF2*. Tubulin was used as the loading control. The result shown is a representative of 3 independent experiments. **b.** Xenograft experiment using inducible *NRF2*-knockdown and control H460 cells (n=8 each; number of xenograft tumors). 1 x 10^4 cells were mixed with Matrigel and subcutaneously transplanted into nude mice. Tumors were weighed after 21 days. A photograph shows xenograft tumors at the time of tumor weight measurement. Horizontal bars indicate median levels (middle panel). Data are presented as mean + SEM (left panel). Two-sided Wilcoxon rank sum test was performed. $^{**}p<0.005$. **c.** RT-PCR measuring the expression of stem cell marker genes normalized to *HPRT* in parental and oncosphere-forming A549 cells. The average and SD of 3 independent experiments are shown. Two-sided Student's *t* test was performed. $^{*}p<0.05$, $^{**}p<0.005$.

Supplementary Fig. 2

a

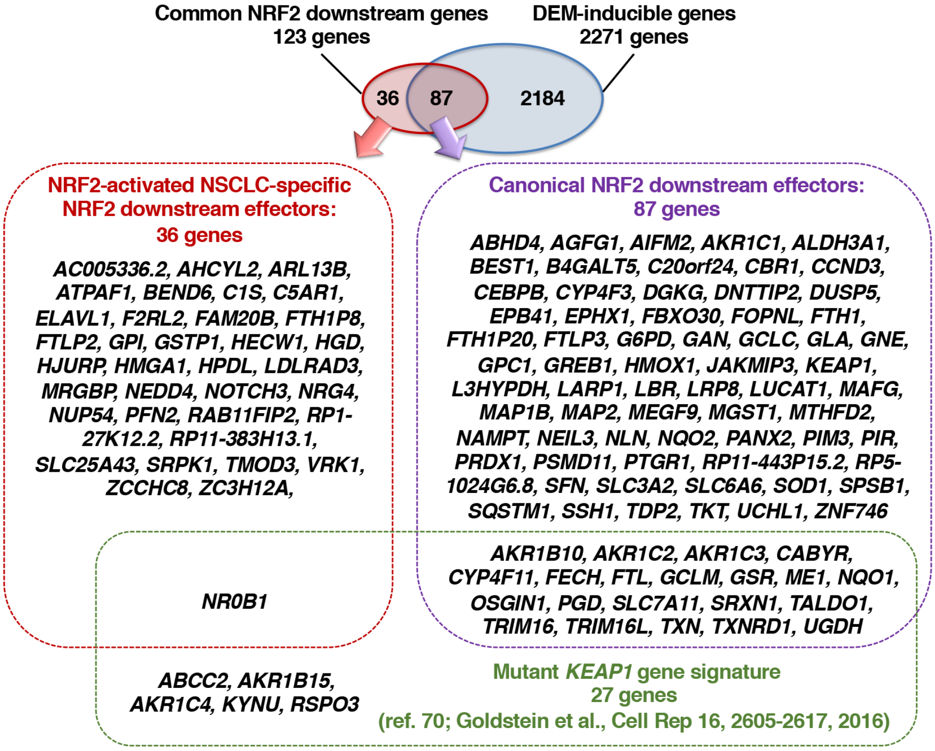

b

Correlation with NRF2 score#	Correlation coefficient < 0.2	Correlation coefficient > 0.2
Genes	*AHCYL2*<br>*ARL13B*<br>*ATPAF1*<br>*BEND6*<br>*C1S*<br>*C5AR1*<br>*ELAVL1*<br>*HECW1*<br>*LDLRAD3*<br>*MRGBP*<br>*NEDD4*<br>*NUP54*<br>*RAB11FIP2*<br>*SLC25A43*<br>*TMOD3*<br>*ZCCHC8*<br>*ZC3H12A*	***F2RL2***<br>***FAM20B***<br>***GPI***<br>***GSTP1***<br>***HGD***<br>***HJURP***<br>***HMGA1***<br>***HPDL***<br>***NOTCH3***<br>***NRG4***<br>***PFN2***<br>***SRPK1***<br>***VRK1***

#NRF2 score: Average value of z-scores of 6 representative NRF2 target genes, *NQO1*, *SLC7A11*, *GCLC*, *GCLM*, *TXNRD1* and *NR0B1* in LUAD data in TCGA, PanCancer Atlas (503 samples).

c

A549

Cell proliferation	Decreased compared with Control (p value <0.05)	Not decreased
Genes	*F2RL2*<br>*GPI*<br>*HJURP*<br>*HGD*<br>*HPDL*<br>*PFN2*<br>*SRPK1*<br>*VRK1*	***FAM20B***<br>***GSTP1***<br>***HMGA1***<br>***NRG4***<br>***NOTCH3***

H460

Cell proliferation	Decreased compared with Control (p value <0.05)	Not decreased
Genes	*F2RL2*<br>*FAM20B*<br>*GPI*<br>*GSTP1*<br>*HGD*<br>*HJURP*<br>*HMGA1*<br>*HPDL*<br>*NRG4*<br>*PFN2*<br>*SRPK1*	***NOTCH3***<br>***VRK1***

H2023

Cell proliferation	Decreased compared with Control (p value <0.05)	Not decreased
Genes	*F2RL2*<br>*GPI*<br>*HJURP*<br>*HMGA1*<br>*HPDL*<br>*NRG4*<br>*PFN2*<br>*SRPK1*<br>*VRK1*	***FAM20B***<br>***GSTP1***<br>***HGD***<br>***NOTCH3***

N = 3~6

Supplementary Figure 2. NRF2 downstream effectors identified in RNA-seq analysis with NRF2-activated and NRF2-normal NSCLC cell lines.
a. 87 canonical NRF2 downstream effectors and 36 NRF2-activated cancer-specific NRF2 downstream effectors were compared with the mutant *KEAP1* gene signature[70]. **b.** Correlations with NRF2 activity were examined for the 36 NRF2-activated cancer-specific NRF2 downstream effector genes using LUAD transcriptome data registered in TCGA database. NRF2 activity was expressed as NRF2 score, which was defined as an average value of z-scores of 6 representative NRF2 target genes, *NQO1*, *SLC7A11*, *GCLC*, *GCLM*, *TXNRD1* and *NR0B1*. Correlation coefficient was calculated for an z-score of each gene, and genes exhibiting correlation coefficient larger than 0.2 were selected because the correlation coefficient 0.2 is sufficiently statistically significant by two-sided test for the sample size as 503. **c.** Impacts on cell proliferation of NRF2-activated NSCLC cell lines were examined for 13 genes selected in panel **b**. For all three NSCLC cell lines, *NOTCH3* was included in a group of genes whose inhibition did not suppress the proliferation measured by spheroid growth. Spheroid growth was examined in 3 (A549) and 6 (H460, H2023) independent experiments with each siRNA. Two-sided Student's *t* test was performed.

Supplementary Fig. 3

a

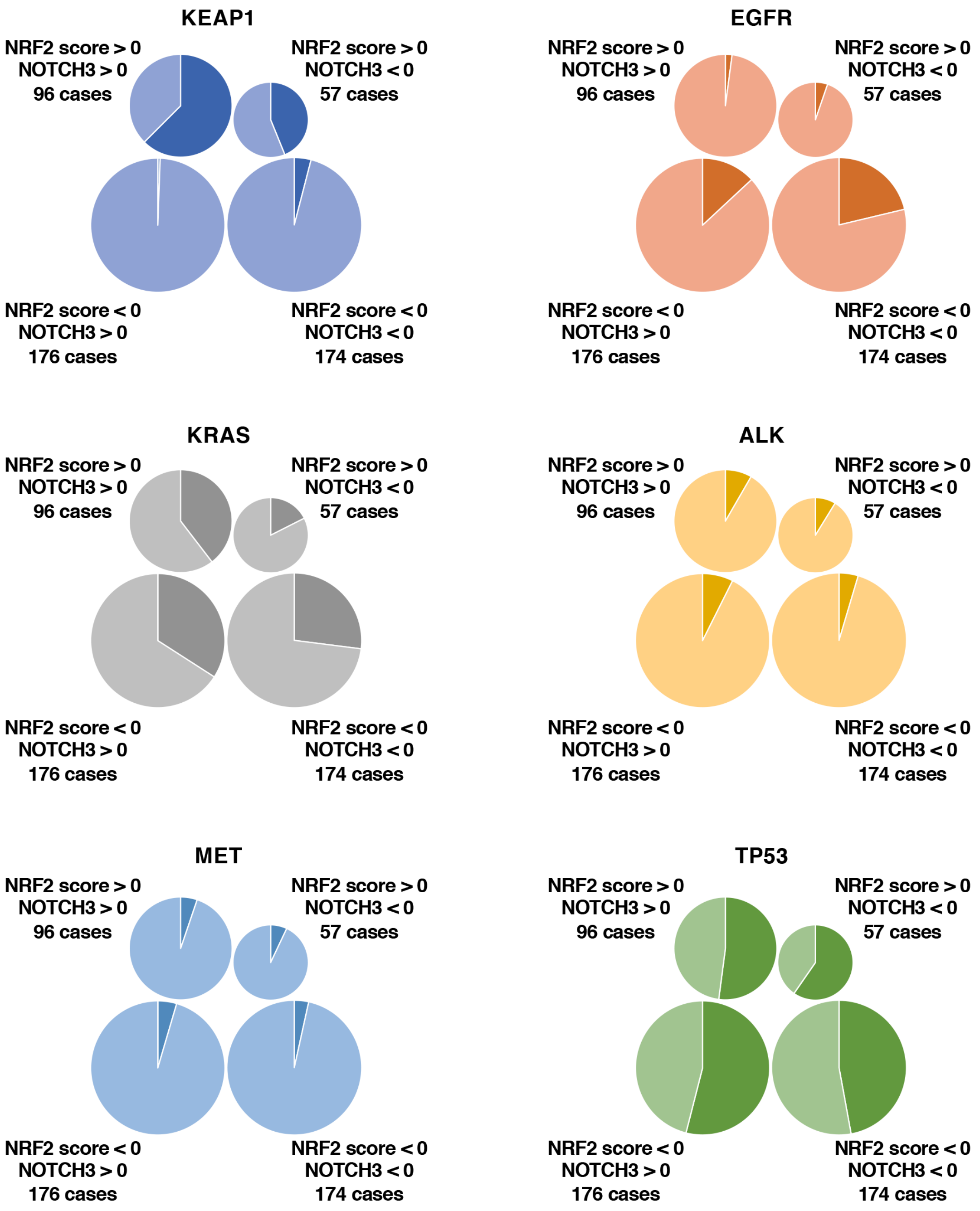

b

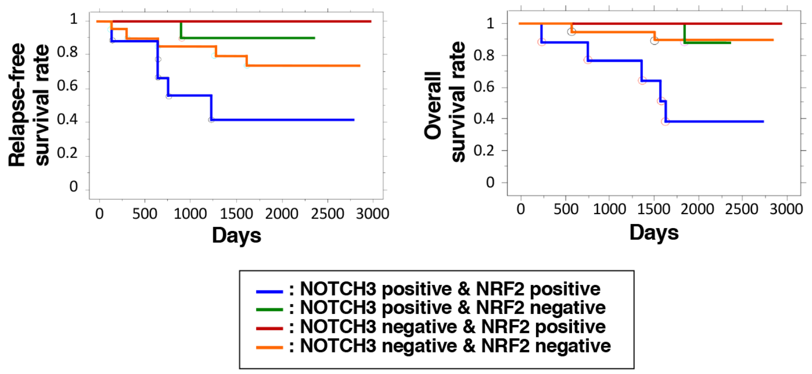

Supplementary Figure 3. Relation between *NRF2* and *NOTCH3* in clinical samples.
a. Mutation ratios of *KEAP1* and other representative oncogenes in LUAD samples registered in TCGA database, which were divided into 4 groups according to *NOTCH3* mRNA level expressed as z-score and NRF2 activity expressed as NRF2 score (see a legend to Supplementary Figure 2**b**). Pie chart slices in dark color indicate proportions of the cases with respective gene mutations. **b.** Overall survival rates and relapse-free survival rates of LUAD patients according to NRF2 and NOTCH3 statuses are shown.

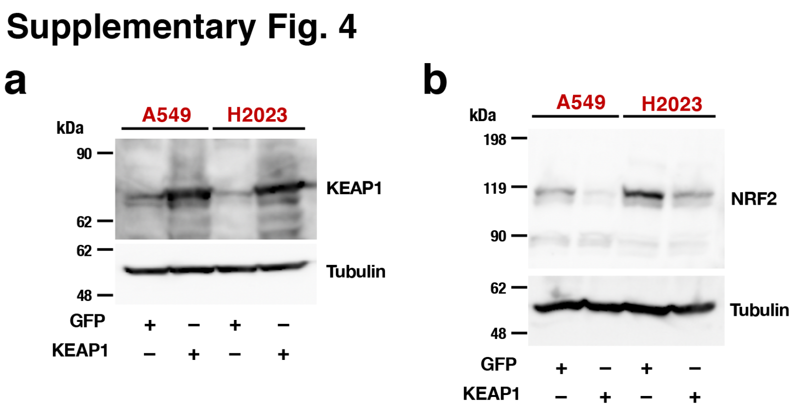

Supplementary Figure 4. Reconstitution of wild-type KEAP1 in NRF2-activated NSCLC cell lines.
a,b. Immunoblot analysis detecting KEAP1 (**a**) and NRF2 (**b**) in NRF2-acivated NSCLC cell lines with GFP expression (control) or wild-type KEAP1 reconstitution. Tubulin expression was used as a loading control. The results shown are representative of 3 independent experiments.

Supplementary Fig. 5

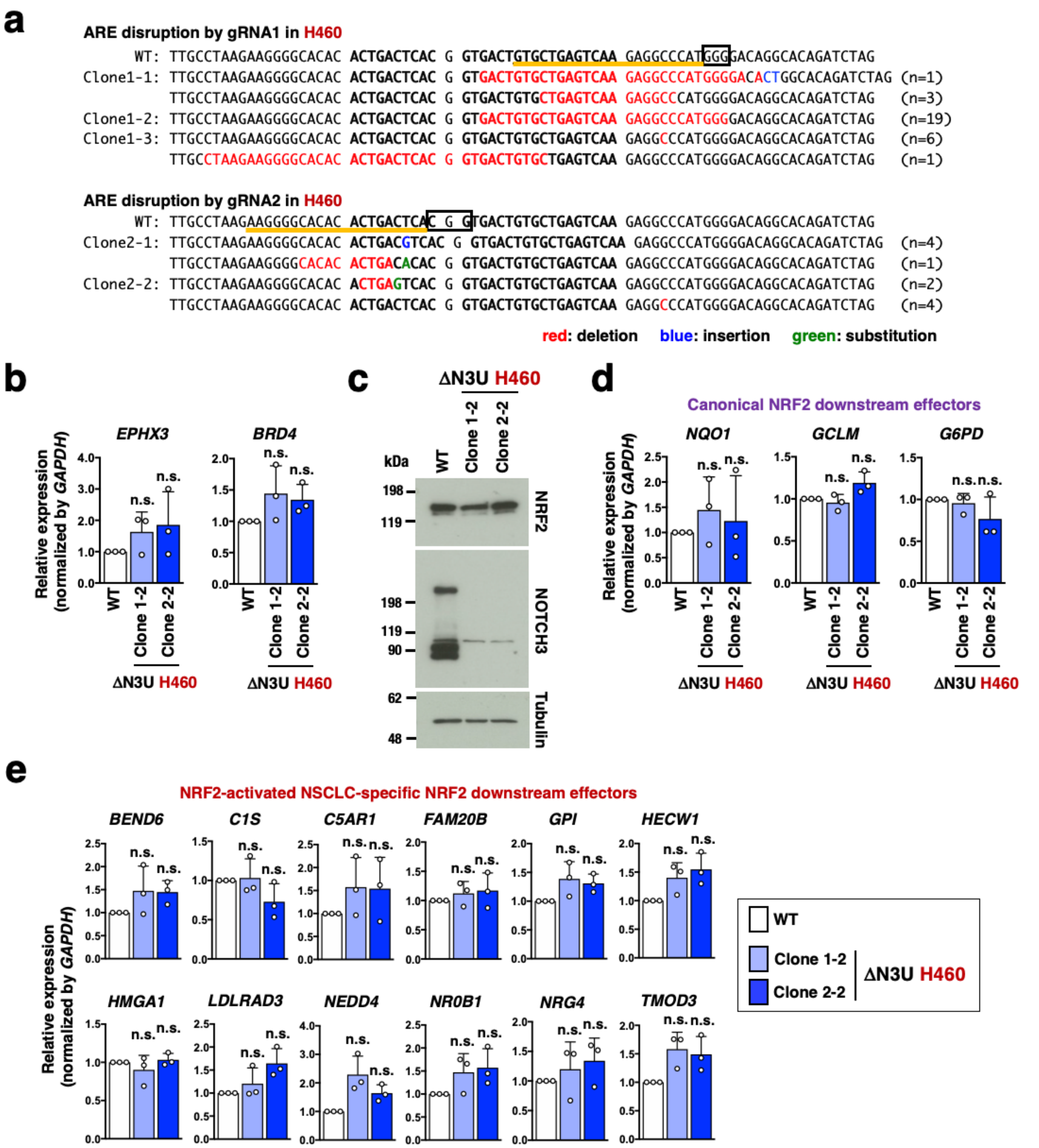

Supplementary Figure 5. Genome editing of the *NOTCH3* upstream region in H460 cells. **a.** DNA sequences of NRF2-binding regions were altered by CRISPR-Cas9 genome editing in the *NOTCH3* enhancer of H460. Sequences corresponding to gRNAs are underlined in orange, and protospacer-adjacent motifs are boxed. PCR products were cloned and sequenced using primer pairs flanking the targeted regions. Deleted, inserted and substituted bases are indicated in red, blue and green, respectively. Numbers of altered sequences obtained in the cloned PCR products are shown to the right of the figure. **b,d,e.** RT-PCR measuring the expression of genes neighboring *NOTCH3* (**b**), canonical NRF2 downstream effector genes (**d**) and NRF2-activated NSCLC-specific NRF2 downstream effector genes (**e**) normalized to *GAPDH* in ΔN3U and WT H460 cells. Fold changes

of the normalized values were calculated in comparison to WT H460 cells. The average and SD of the fold changes from 3 independent experiments are shown. Comparison was made between two cell groups; WT H460 and each mutant clone. Two-sided confidence interval estimation was conducted for the ΔN3U H460 clones. n.s.: not significant. **c.** Immunoblot analysis of NOTCH3 and NRF2 protein expression. Tubulin expression was used as a loading control. The results shown are representative of 3 independent experiments.

Supplementary Fig. 6

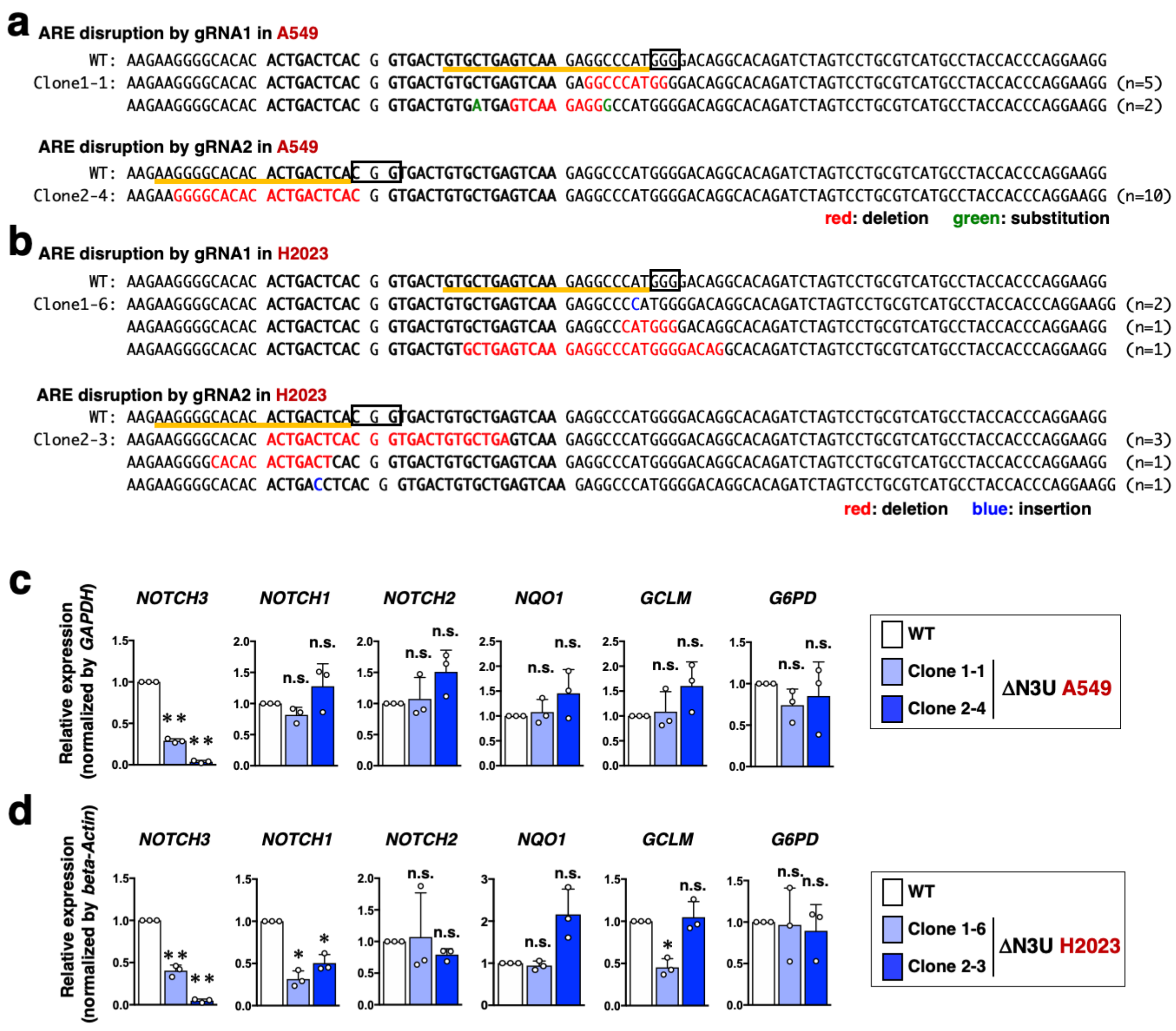

Supplementary Figure 6. Genome editing of the *NOTCH3* upstream region in A549 and H2023 cells.

a,b. DNA sequences of NRF2-binding regions were altered by CRISPR-Cas9 genome editing in the *NOTCH3* enhancer of A549 (**a**) and H2023 (**b**) cells. Sequences corresponding to gRNAs are underlined in orange, and protospacer-adjacent motifs are boxed. PCR products were cloned and sequenced using primer pairs flanking the targeted regions. Deleted, inserted and substituted bases are indicated in red, blue and green, respectively. Numbers of altered sequences obtained in the cloned PCR products are shown to the right of the figure. **c,d.** RT-PCR measuring the expression of the *NOTCH* family and canonical NRF2 target genes in ΔN3U and WT A549 cells normalized to *GAPDH* (**c**) and ΔN3U and WT H2023 cells normalized to *beta-Actin* (**d**). Fold changes of the normalized values were calculated in comparison to WT A549 and WT H2023 cells. The average and SD of the fold changes from 3 independent experiments are shown. Comparison was made between two cell groups; WT (A549 or H2023) and each mutant clone. Two-sided confidence interval estimations were conducted for the ΔN3U A549 and ΔN3U H2023 clones. * $\alpha<0.05$, ** $\alpha<0.01$, n.s.: not significant.

Supplementary Fig. 7

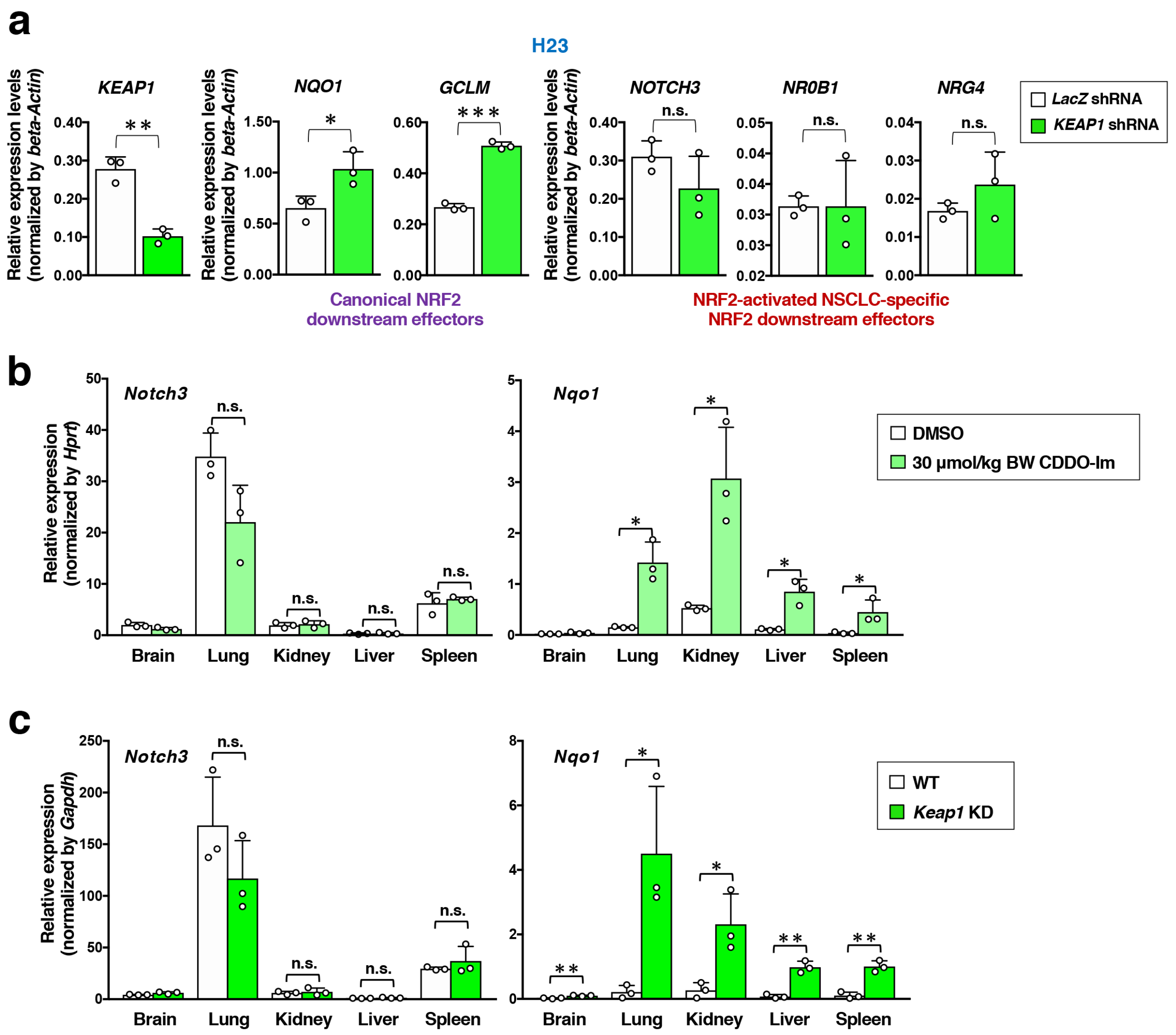

Supplementary Figure 7. Impacts of genetic and pharmacological activation of NRF2 on NRF2 downstream effectors in NRF2-normal NSCLC cell line and normal mouse tissues.
a. RT-PCR measuring the expression of canonical and NRF2-activated NSCLC-specific NRF2 downstream effectors normalized by *beta-Actin* in H23, one of NRF2-normal NSCLC cell line, with or without *KEAP1* knockdown. **b.** RT-PCR measuring the expression of *Notch3,* one of NRF2-activated NSCLC-specific NRF2 downstream effectors, and *Nqo1,* one of canonical NRF2 downstream effectors, normalized to *Hprt* in representative tissues of mice treated with vehicle (DMSO) or CDDO-Im. DMSO; dimethyl sulfoxide, CDDO-Im; 2-Cyano-3,12-dioxooleana-1,9(11)-dien-28-oic acid imidazolide. **c.** RT-PCR measuring the expression of *Notch3* and *Nqo1* normalized to *Gapdh* in representative tissues of WT and *Keap1* knockdown mice.
The average and SD of 3 independent experiments are shown. Two-sided Student's *t* test was performed. **p*<0.05, ***p*<0.005, ****p*<0.0005, n.s.: not significant.

Supplementary Fig. 8

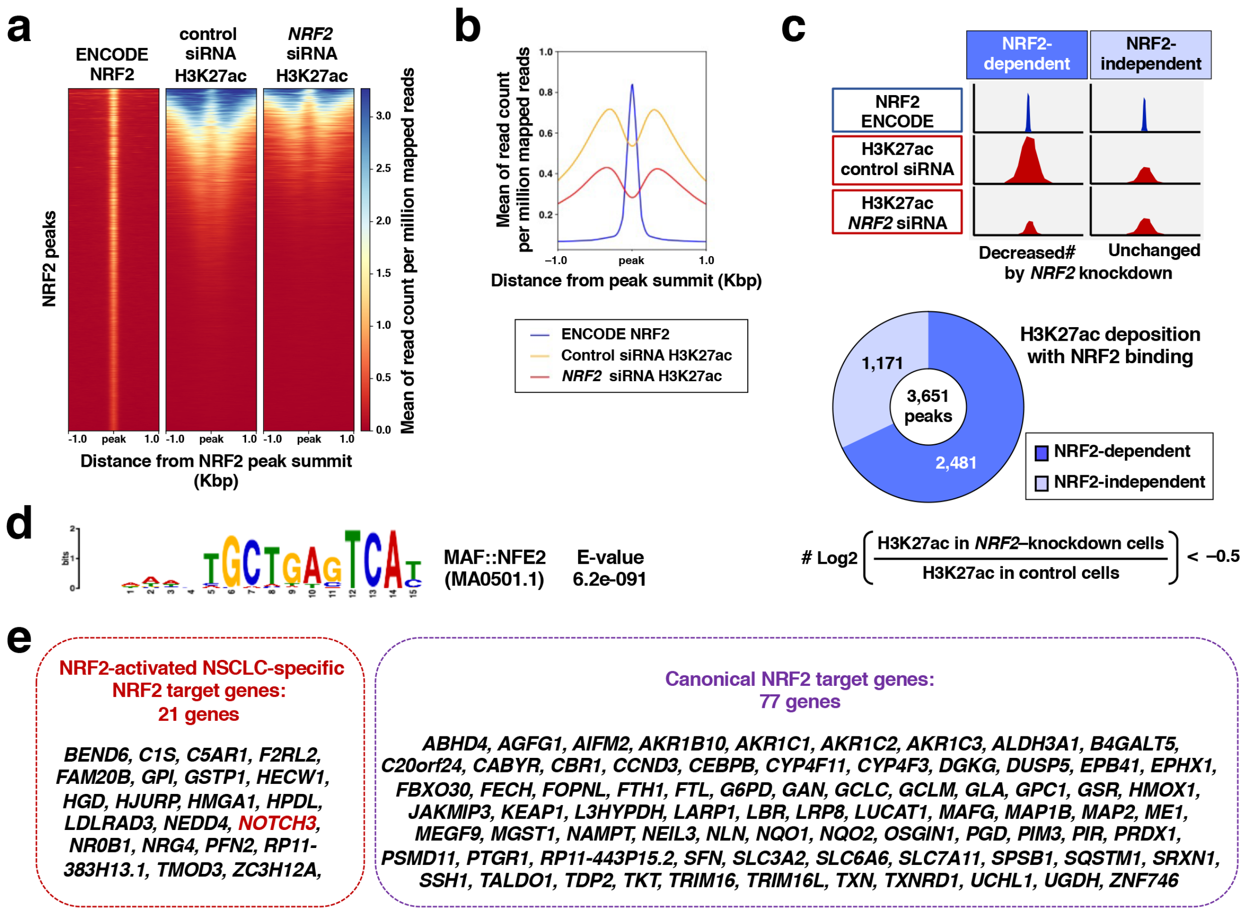

Supplementary Figure 8. NRF2 contributes to unique enhancer formation in NRF2-activated NSCLC cells.
a,b. ChIP-seq analysis using an antibody against H3K27ac in A549 cells treated with control siRNA or *NRF2* siRNA. A heat map (**a**) and an aggregation plot (**b**) show acetylated H3K27 (H3K27ac) deposition surrounding NRF2 binding sites obtained from the ENCODE database. **c.** Classification of NRF2-bound enhancers according to the NRF2 dependency of H3K27ac deposition. **d.** A motif enriched in NRF2-dependent enhancers obtained using the MEME-ChIP platform. **e.** NRF2-activated NSCLC-specific NRF2 target genes and canonical NRF2 target genes based on the presence of NRF2-dependent enhancers.

Supplementary Fig. 9

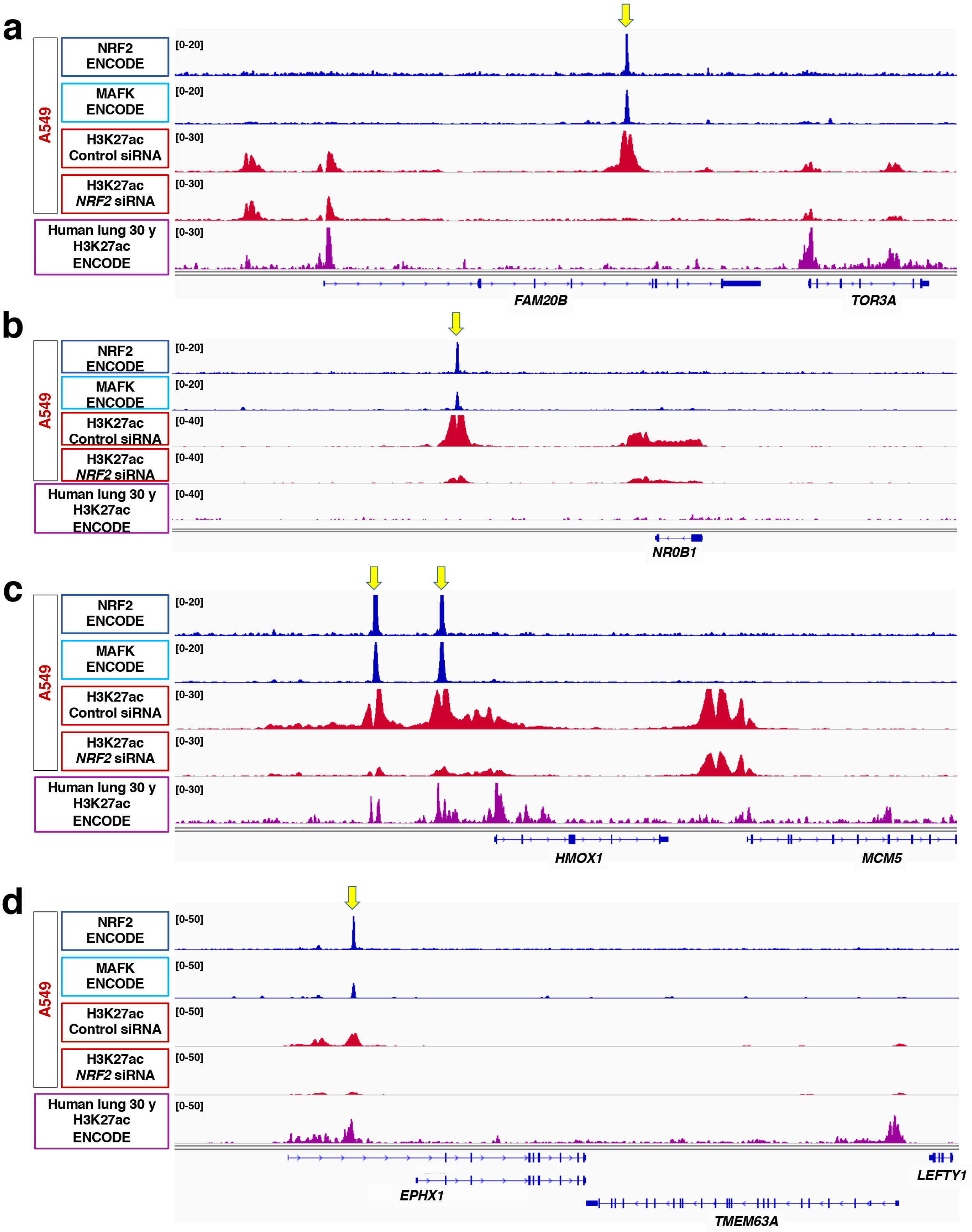

Supplementary Figure 9. ChIP-seq profiles at representative NRF2-activated NSCLC-specific and canonical NRF2 target loci.
Genome browser views of NRF2-activated NSCLC-specific NRF2 target loci (**a**,**b**) and canonical NRF2 target loci (**c**,**d**). A549 cells (upper panels) and normal human lung samples (lower panel) are shown. NRF2 and MAFK chromatin binding in A549 cells and H3K27ac deposition patterns in

normal human lung samples were obtained from the ENCODE database. Acetylated H3K27 deposition profiles in A549 cells treated with control siRNA or *NRF2* siRNA were obtained in this study. Yellow arrows indicate NRF2 binding peaks in the respective NRF2 target loci.

Supplementary Fig. 10

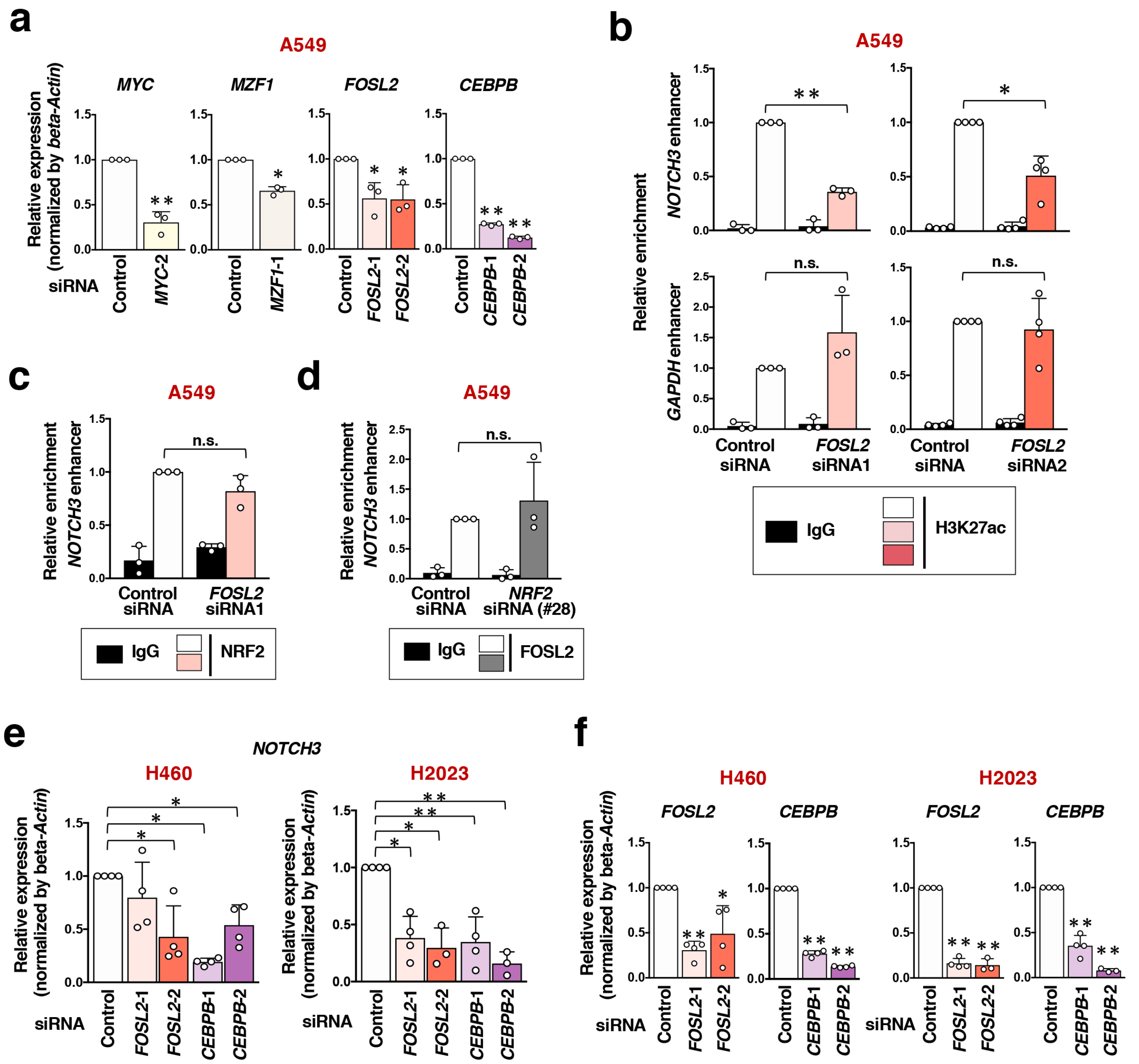

Supplementary Figure 10. CEBPB cooperates with NRF2 and contributes to *NOTCH3* enhancer formation in NRF2-activated NSCLCs.

a. RT-PCR measuring the knockdown efficiency of siRNAs against *MYC*, *MZF1*, *FOSL2* and *CEBPB* in A549 cells. Fold changes of the normalized values were calculated in comparison to control siRNA-treated cells. The average and SD of the fold changes from 3 independent experiments are shown. Comparison was made between two cell groups; control siRNA and each test siRNA. Two-sided confidence interval estimation was conducted to evaluate statistical significance. * $\alpha<0.05$, ** $\alpha<0.01$. **b,c.** ChIP assay using H3K27ac (**b**) and NRF2 (**c**) antibodies in A549 cells treated with *FOSL2* or control siRNAs. Enrichment of the *NOTCH3* enhancer region was examined. The *GAPDH* enhancer was selected as a control locus (**b**). Fold changes of %input values were calculated in comparison to the control cells reacted with H3K27ac or NRF2 antibody. The average and SD of 3 independent experiments are shown except for the experiment with *FOSL2* siRNA2 in panel **b**, which was independently conducted for 4 times. Two-sided confidence interval estimation was conducted for knockdown samples reacted with H3K27ac or NRF2 antibody. * $\alpha<0.05$, n.s.: not significant. **d.** ChIP assay using FOSL2 antibody in A549 cells treated

with NRF2 or control siRNAs. Enrichment of the *NOTCH3* enhancer region was examined. Fold changes of %input values were calculated in comparison to the control cells reacted with FOSL2 antibody. The average and SD of 3 independent experiments are shown. Two-sided confidence interval estimation was conducted for knockdown samples reacted with FOSL2 antibody. n.s.: not significant. **e.** RT-PCR measuring the expression of *NOTCH3* normalized to *beta-Actin* in H460 and H2023 cells treated with *FOSL2*, *CEBPB* and control siRNAs. Fold changes of the normalized values were calculated in comparison to control siRNA-treated cells. The average and SD of the fold changes from 4 independent experiments are shown except for H2023 cells treated with *FOSL2*-2, *CEBPB*-2, which were examined in 3 independent experiments. Comparison was made between two cell groups; control siRNA and each test siRNA. Two-sided confidence interval estimation was conducted to evaluate statistical significance. * $\alpha<0.05$, ** $\alpha<0.01$. **f.** RT-PCR measuring the knockdown efficiency of siRNAs against *FOSL2* and *CEBPB* in H460 and H2023 cells. Fold changes of the normalized values were calculated in comparison to control siRNA-treated cells. The average and SD of the fold changes from 4 independent experiments are shown. Comparison was made between two cell groups; control siRNA and each test siRNA. Two-sided confidence interval estimation was conducted to evaluate statistical significance. * $\alpha<0.05$, ** $\alpha<0.01$.

Supplementary Fig. 11

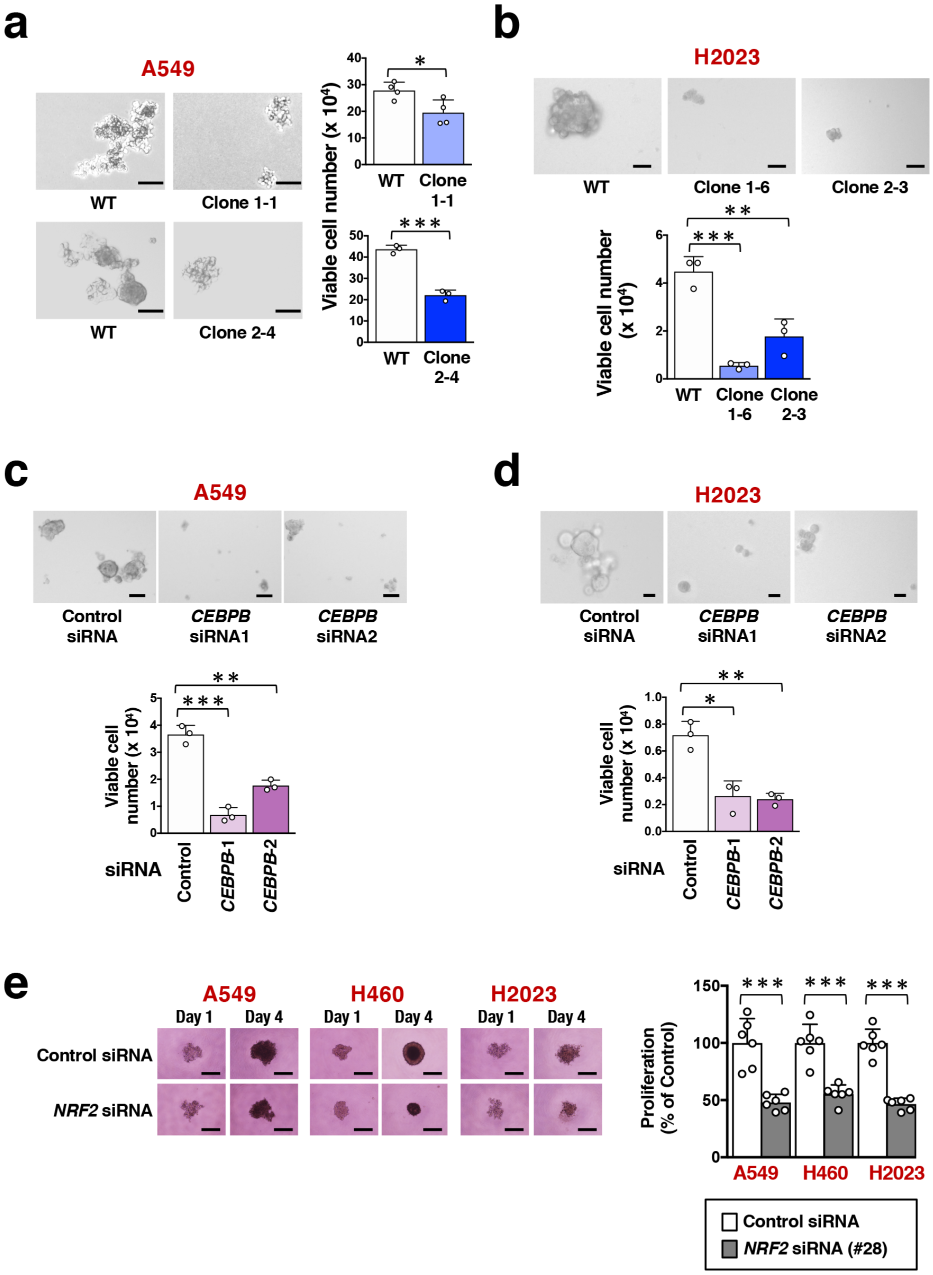

Supplementary Figure 11. ***NOTCH3*** **enhancer promotes oncosphere growth of NRF2-activated NSCLC cell lines.**

a. Oncosphere growth of ΔN3U and WT A549 cells (left panels). Scale bars indicate 200 μm. Viable cells were counted after trypsinization (right panels). Average cell numbers and SD from 3 (Clone 2-4) and 4 (Clone 1-1) independent experiments are shown. Two-sided Student's *t* test was performed. **b.** Oncosphere growth of ΔN3U and WT H2023 cells (upper panels). Scale bars indicate 100 μm. Viable cells were counted after trypsinization (lower panel). Average cell numbers and SD from 3 independent experiments are shown. Comparison was made between two cell groups; WT H2023 and each mutant clone. Two-sided Student's *t* test was performed. **c,d.** Oncosphere growth of A549 (**c**) and H2023 (**d**) with or without *CEBPB* knockdown (upper panels). Scale bars indicate 100 μm (**c**) and 50 μm (**d**). Viable cells were counted after trypsinization (lower panels). Average cell numbers and SD from 3 independent experiments are shown. Comparison was made between

two cell groups; control siRNA and each test siRNA. Two-sided Student's *t* test was performed. **e.** Spheroid growth of A549, H2023 and H460 cells. Spheroids are shown at 1 and 4 days after transfection of *NRF2* siRNA (left panels). Scale bars indicate 100 µm. Cell numbers were estimated using a cell counting kit on day 4 (right panel). Average cell numbers and SD from 6 independent experiments are shown. The average cell numbers from NSCLC cell lines treated with control siRNA were set as 100%. Two-sided Student's *t* test was performed. **p*<0.05, ***p*<0.005, ****p*<0.0005.

Supplementary Fig. 12

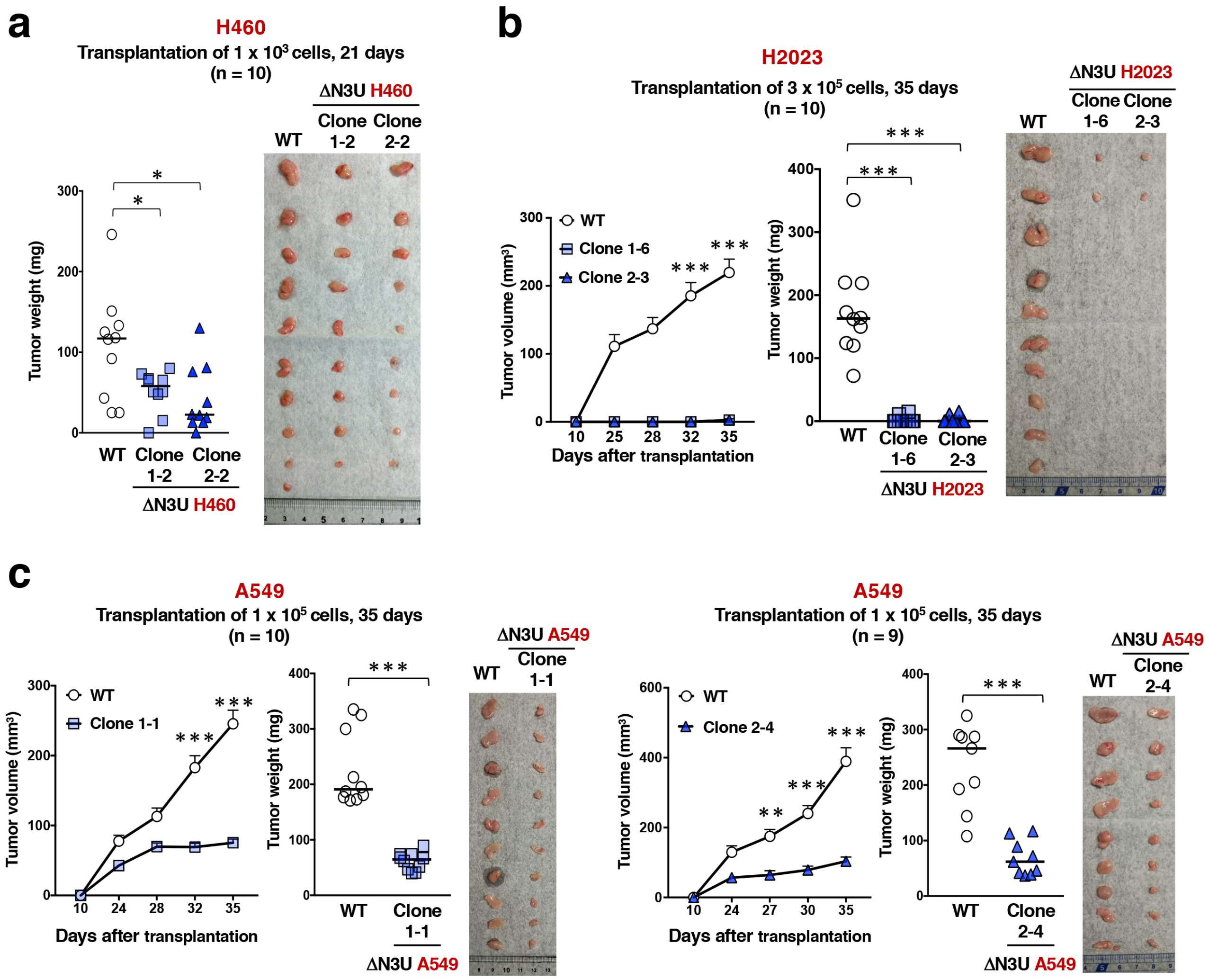

Supplementary Figure 12. ***NOTCH3*** **enhancer promotes tumor-initiating activity of NRF2-activated NSCLCs.**

a. Xenograft experiment using ΔN3U and WT H460 cells (n=10 each; number of xenograft tumors). 1 x 10^3 cells were mixed with Matrigel and subcutaneously transplanted into nude mice. Tumors were weighed after 21 days. A photograph shows xenograft tumors at the time of tumor weight measurement. Horizontal bars indicate the median tumor weight (left panel). Two-sided Wilcoxon rank sum test was performed. **b,c.** Xenograft experiments using ΔN3U and WT H2023 cells (n=10 each; number of xenograft tumors) (**b**) and A549 cells (n=10 for number of xenograft tumors of Clone 1-1, n=9 for those of Clone 2-4) (**c**). 3 x 10^5 (**b**) and 1 x 10^5 cells (**c**) were mixed with Matrigel and subcutaneously transplanted into nude mice. Tumors were weighed after 35 days in both cells. Photographs show xenograft tumors at the time of tumor weight measurement. Horizontal bars indicate the median tumor weight (middle panel). Data are presented as mean + SEM (left panel). Two-sided Wilcoxon rank sum test was performed. *p<0.05, **p<0.005, ***p<0.0005.

Supplementary Fig. 13

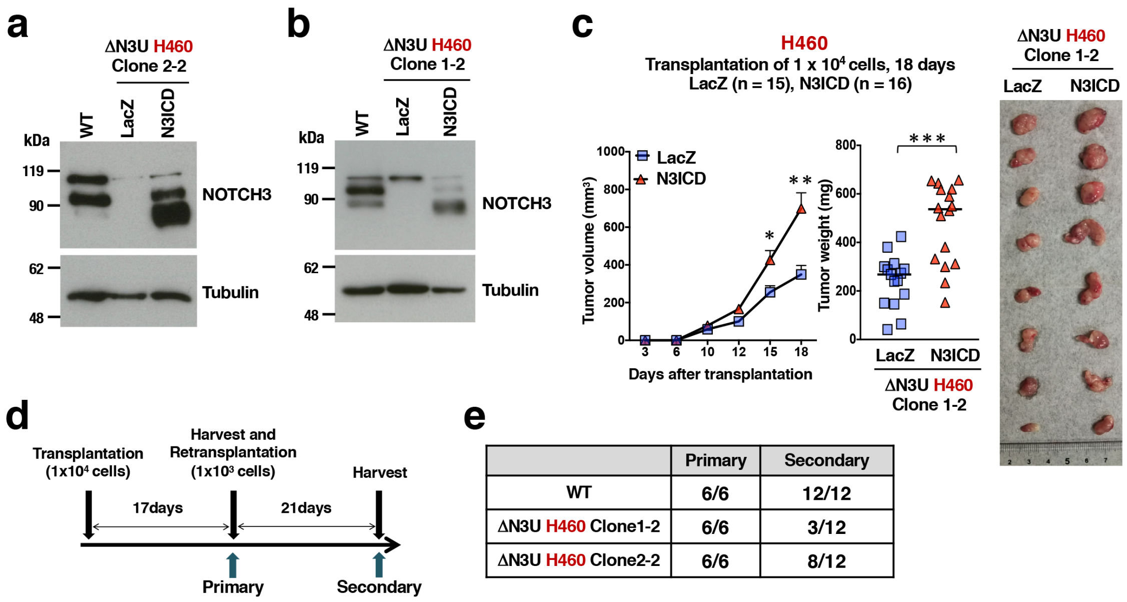

Supplementary Figure 13. Xenograft experiments using ΔN3U H460 cells.
a,b. Immunoblot analysis of NOTCH3 protein levels of WT H460 cells and ΔN3U H460 Clone 2-2 cells (**a**), Clone 1-2 cells (**b**) with LacZ and N3ICD expression. Tubulin expression was used as a loading control. The results shown are representative of three independent experiments. **c.** Xenograft experiment using ΔN3U H460 Clone 1-2 cells expressing LacZ (n=15; number of xenograft tumors) and N3ICD (n=16; number of xenograft tumors). 1 x 10^4 cells of Clone 1-2 were mixed with Matrigel and subcutaneously transplanted into nude mice. Tumors were weighed after 18 days. A photograph shows representative xenograft tumors at the time of tumor weight measurement. Horizontal bars indicate the median tumor weight (middle panel). Data are presented as mean + SEM (left panel). Two-sided Wilcoxon rank sum test was performed. *p<0.05, **p<0.005, ***p<0.0005. **d.** Protocol for the serial transplantation experiment. **e.** Tumor numbers in the primary and secondary transplantation.

Supplementary Figure 14: Uncropped data 1

Supplementary Fig. 14

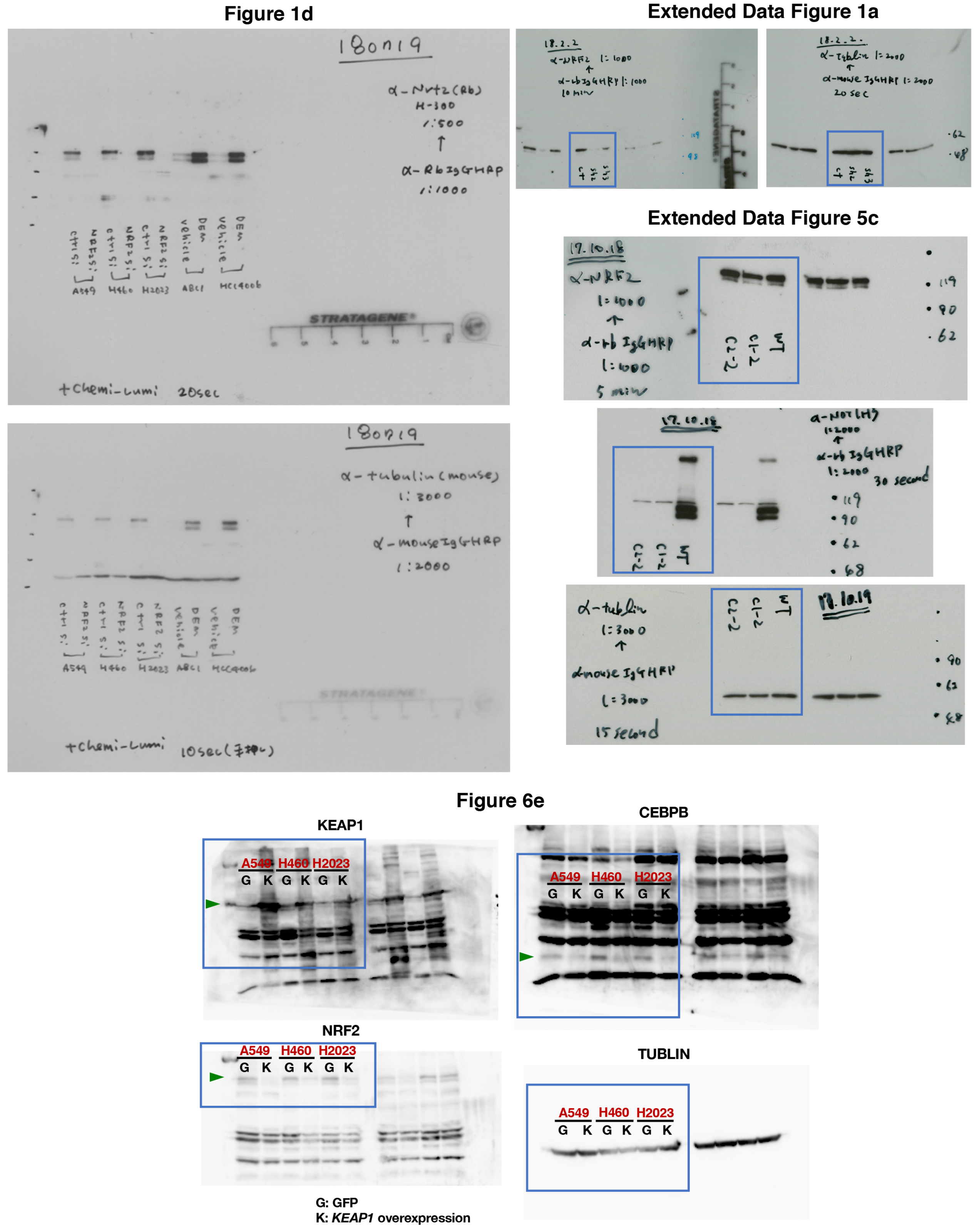

Supplementary Figure 15: Uncropped data 2

Supplementary Fig. 15

Figure 6c

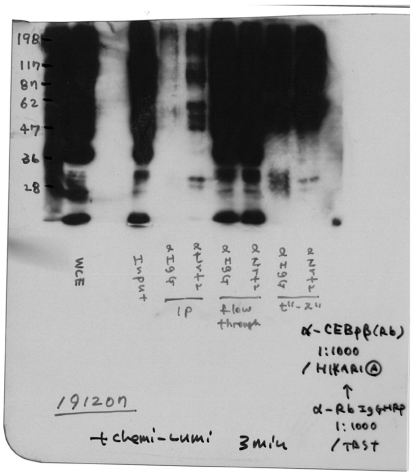

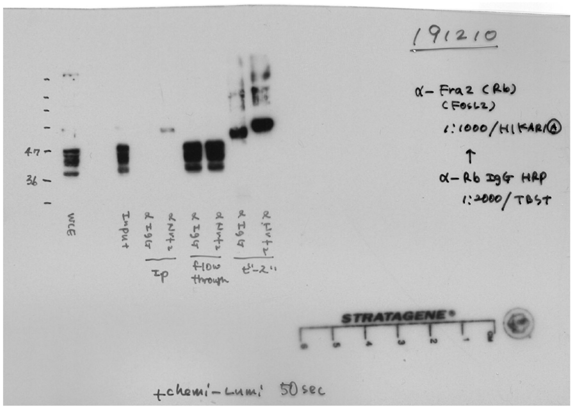

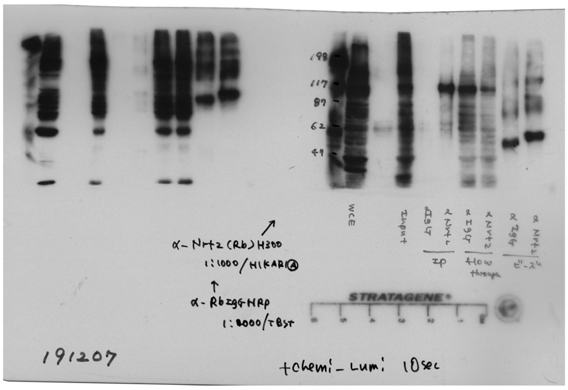

Figure 6d

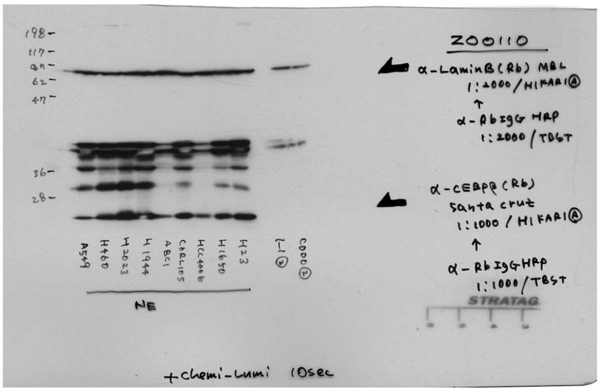

Figure 7a

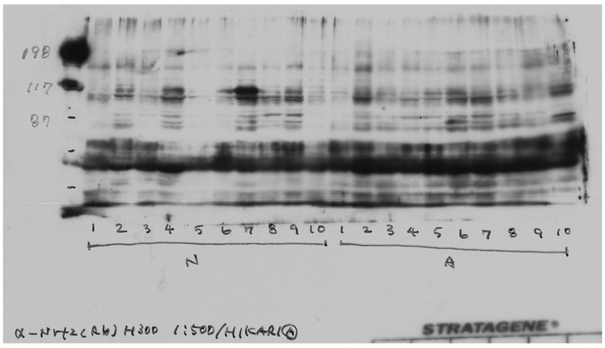

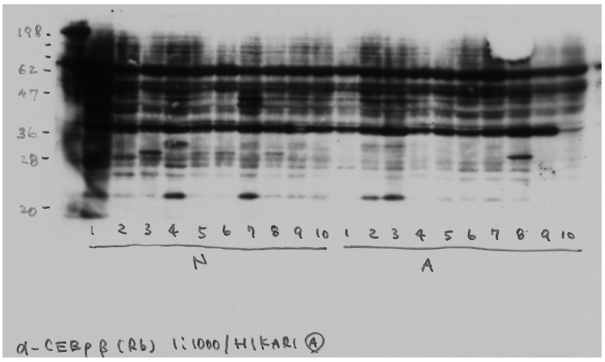

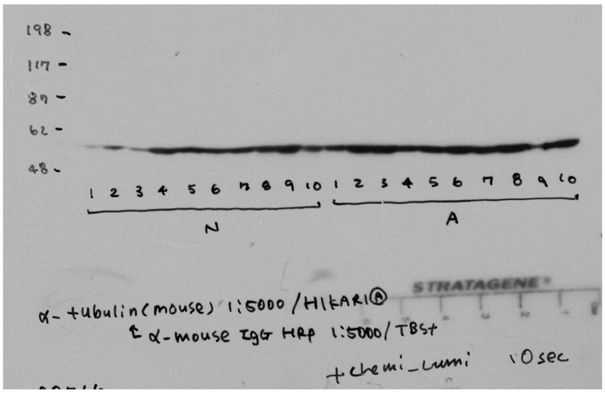

Supplementary Figure 16: Uncropped data 3

Supplementary Fig. 16

Extended Data Figure 4

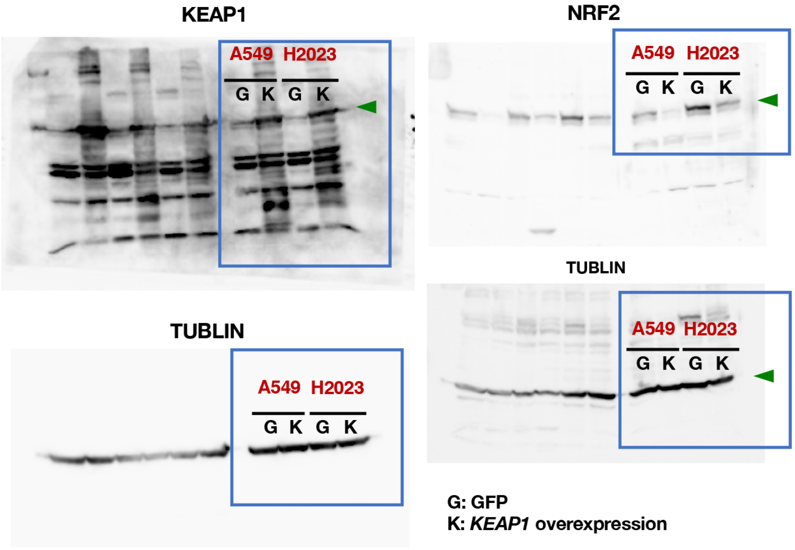

Extended Data Figure 13a (upper panels), b (lower panel)

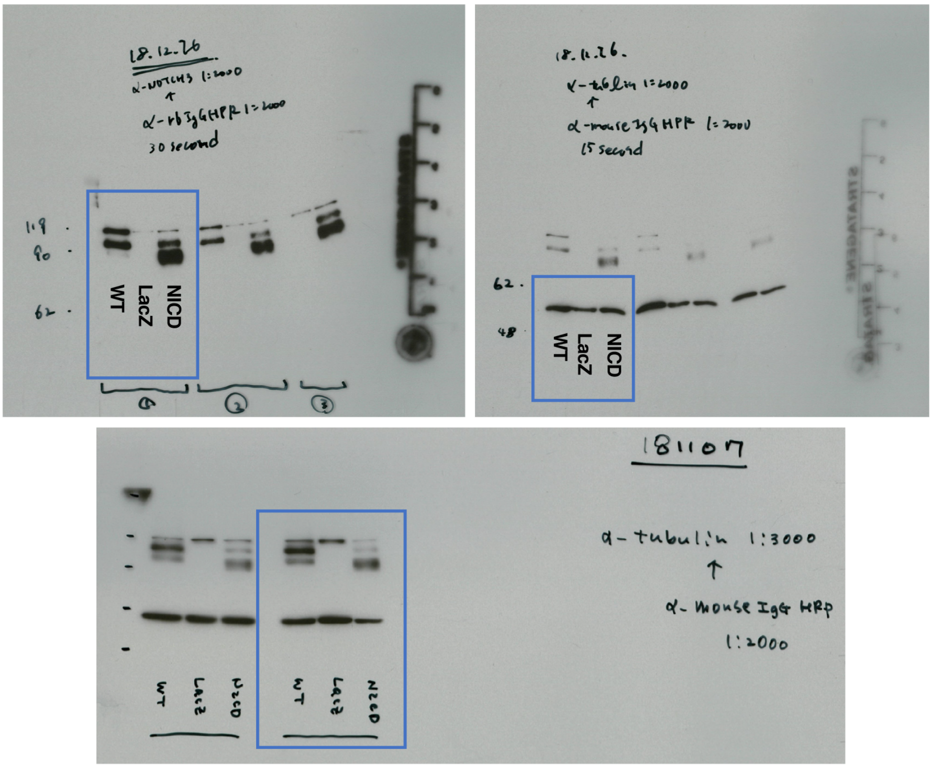

Supplementary Table 1. Genome-wide colocalization with NRF2 and other transcription factors in A549 cells in the basal condition by evaluated Jaccard test.

accession	target	database	with Enhancer (with H3K27ac)	without Enhancer (without H3K27ac)	with Enhancer (rank)	without Enhancer (rank)	jaccard.measure.p.value
ENCSR000BTI	GATA3	ENCODE	0.033744757	0.001995699	1	7	<0.01
ENCSR541WQI	MAFK	ENCODE	0.033241356	0.022426389	2	1	<0.01
ENCSR000BTN	PBX3	ENCODE	0.030213183	0.001062288	3	15	<0.01
**ENCSR000BUB**	**CEBPB**	**ENCODE**	**0.028322256**	**0.002093852**	4	5	<0.01
ENCSR000BUD	TEAD4	ENCODE	0.028189778	0.000711447	5	21	<0.01
ENCSR000DYG	MAX	ENCODE	0.022531067	0.002342195	6	4	<0.01
ENCSR000DYC	MYC	ENCODE	0.019267867	0.001574365	7	11	<0.01
ENCSR897MYK	SREBF1	ENCODE	0.017813202	0.001478614	8	13	<0.01
ENCSR473SUA	ESRRA	ENCODE	0.01774824	0.001004015	9	17	<0.01
ENCSR563FBT	USF2	ENCODE	0.016692417	0.002837884	10	3	<0.01
ENCSR513XQX	SIN3A	ENCODE	0.015571418	0.001627406	11	10	<0.01
ENCSR000BTC	E2F6	ENCODE	0.015021909	0.001511104	12	12	<0.01
ENCSR000DYJ	BHLHE40	ENCODE	0.01460982	0.001841074	13	9	<0.01
ENCSR067HGI	CHD2	ENCODE	0.012152239	0.00037152	14	30	<0.01
ENCSR064LJN	RFX5	ENCODE	0.01150689	0.001184237	15	14	<0.01
ENCSR636YLV	MAZ	ENCODE	0.011176716	0.001054051	16	16	<0.01
ENCSR000BUC	RAD21	ENCODE	0.008558371	0.001891745	17	8	<0.01
ENCSR892DRK	REST	ENCODE	0.008325527	0.000657296	18	25	<0.01
ENCSR550SCU	CHD4	ENCODE	0.007728209	0.000750546	19	20	<0.01
ENCSR541AOQ	PHF8	ENCODE	0.00732568	0.000667458	20	24	<0.01
ENCSR481YWD	SMC3	ENCODE	0.006154658	0.003526014	21	2	<0.01
ENCSR996DUT	JUN	ENCODE	0.005716291	0.000220092	22	32	<0.01
ENCSR933MHJ	KDM5A	ENCODE	0.004720808	0.000705754	23	22	<0.01
ENCSR294JWV	ZFP36	ENCODE	0.004332277	0.000753228	24	19	<0.01
ENCSR639GWS	KDM1A	ENCODE	0.004290807	0.00038596	25	29	<0.01
ENCSR638QYO	SREBF2	ENCODE	0.003577102	0.000499624	26	26	<0.01
ENCSR659LJJ	HDAC2	ENCODE	0.003548407	0.000678257	27	23	<0.01
ENCSR000DYD	CTCF	ENCODE	0.003141084	0.002051332	28	6	<0.01
ENCSR623KNM	ELK1	ENCODE	0.002188573	7.06E-05	29	34	<0.01
ENCSR798EGJ	RNF2	ENCODE	0.001414865	0.000767199	30	18	<0.01
ENCSR618ICR	RCOR1	ENCODE	0.001410729	0.000493382	31	27	<0.01
ENCSR321BJQ	EHMT2	ENCODE	0.001028906	0.000118121	32	33	<0.01
ENCSR277OOQ	ZC3H11A	ENCODE	0.000258965	0.000359323	33	31	0.41
ENCSR616MOB	CBX8	ENCODE	0.000254679	0.000418671	34	28	0.37
ENCSR469WAO	CBX2	ENCODE	9.89E-06	0	35	35	0.02

Supplementary Table 2. Primers and probes used for RT-PCR.

Name	Sequence (5'-3')
hKEAP1 forward	CTGGAGGATCATACCAAGCAGG
hKEAP1 reverse	GAACATGGCCTTGAAGACAGG
hNRF2 forward	TCATGATGGACTTGGAGCTG
hNRF2 reverse	CATACTCTTTCCGTCGCTGA
hNQO1 forward	GTCATTCTCTGGCCAATTCAGAGT
hNQO1 reverse	TTCCAGGATTTGAATTCGGG
hNQO1 probe	FAM-ACTGACATATAGCATTGGGCACACTCCAG -TAMRA
hGCLM forward	TAGAATCAAACTCTTCATCATCAACTAGA
hGCLM reverse	TCACAGAATCCAGCTGTGCAA
hGCLM probe	FAM-TGCAGTTGACATGGCCTGTTCAGTCC-TAMRA
hG6PD forward	TGACCTGGCCAAGAAGAAGA
hG6PD reverse	CAAAGAAGTCCTCCAGCTTG
hNANOG forward	ATGCCTCACACGGAGACTG
hNANOG reverse	GTTGTTTGCCTTTGGGACTG
hCD133 forward	CTATTCAGGATATACTCTCAGCATT
hCD133 reverse	TTTCTGTGGATGTAACTTTCAGTG
hOCT3/4 forward	TTCGCAAGCCCTCATTTC
hOCT3/4 reverse	GAGAAGGCGAAATCCGAAG
hNOTCH1 forward	CAATGAGTTCCAGTGCGAGT
hNOTCH1 reverse	GTAAGTGTTGGGTCCGTCCA
hNOTCH2 forward	ACCTACCACAATGGCACAGG
hNOTCH2 reverse	GCAGCGGTTCTTCTCACAG
hNOTCH3 forward	TCATCCGAAACCGCTCTACA
hNOTCH3 reverse	GCTCATCCACAGCATTGACA
hEPHX3 forward	CATGGTTGTGGTCAGTGGTG
hEPHX3 reverse	TGTAGTGGGAACGGAAGAAC
hBRD4 forward	CCACACTGCGTGAGCTGGAG
hBRD4 reverse	ATCTTGGAGGAGCCGGCAAT
hMYC forward	ACGTTAGCTTCACCAACAGGA
hMYC reverse	GTTCTCCTCCTCGTCGCAGTA
hMZF1 forward	AGTGTAAGCCCTCACCTCC
hMZF1 reverse	GGGTCCTGTTCACTCCTCAG
hFOSL2 forward	GAGAGGAACAAGCTGGCTGC
hFOSL2 reverse	GCTTCTCCTTCTCCTTCTGC
hCEBPB forward	CGAAGTTGATGCAATCGGTTT
hCEBPB reverse	TTAAGCGATTACTCAGGGCCC
BEND6 forward	GAGAGACCCATATTCGGGAAA
BEND6 reverse	GCGCACAATTCTTCCTTTGA
C1S forward	AAGTTGAAGACCCAGAGAGCAC
C1S reverse	CTCCCGTTACCAGCACAGT
C5AR1 forward	GACTACAGCCACGACAAACG
C5AR1 reverse	CGGAGCAGGATGAAAGTGT
FAM20B forward	AGAGATCAAACCTGTCGCC
FAM20B reverse	CCAAAGTGTGACAGATCCCT
GPI forward	CGCCCAACCAACTCTATTGT
GPI reverse	AGATGATGCCCTGAACGAAG

HECW1 forward	CAAGGAAGTCGGAGGCTGAT
HECW1 revrese	AGATAAGGGTCTGGGTTGAAA
HMGA1 forward	AAACAAGGGTGCTGCCAAG
HMGA1 reverse	ATGGGTCACTGCTCCTCCT
LDLRAD3 forward	CAGAATGCGTCGGAAGTAGG
LDLRAD3 reverse	AGAAGAGTAGGGCGGTGGAG
NEDD4 forward	TTGAATGCCAGACTCACCAT
NEDD4 reverse	GCACAGGAAGTGTAGGTTGTTC
NR0B1 forward	CTTTCCAAATGCTGGAGTCTG
NR0B1 reverse	ACGTCCGGGTTAAAGAGCAC
NRG4 forward	GGTCCCAGTCACAAGTCGTT
NRG4 reverse	CAATGCCACAAAAGCTTCAA
TMOD3 forward	GGGAAGTAGTAATGGTGTTGACC
TMOD3 reverse	GTTGGATTTGGTGGCTCATC
hACTB forward	TGGCACCCAGCACAATGAA
hACTB reverse	CTAAGTCATAGTCCGCCTAGAAGCA
hGAPDH forward	GAAGGTGAAGGTCGGAGTC
hGAPDH reverse	GAAGATGGTGATGGGATTTC
hGAPDH probe	FAM-CAAGCTTCCCGTTCTCAGCC-TAMRA
hHPRT forward	CCGGCTCCGTTATGGC
hHPRT reverse	GGTCATAACCTGGTTCATCATCA
hHPRT probe	FAM-CGCAGCCCTGGCGTCGTGATTA-TAMRA
mNotch3 forward	AGCTGGGTCCTGAGGTGAT
mNotch3 reverse	AGACAGAGCCGGTTGTCAAT
mNqo1 forward	AGCTGGAAGCTGCAGACCTG
mNqo1 reverse	CCTTTCAGAATGGCTGGCA
mNqo1 probe	FAM-ATTTCAGTTCCCATTGCAGTGGTTTGGG-TAMRA
mHprt forward	CTGGTGAAAAGGACCTCTCG
mHprt reverse	TGAAGTACTCATTATAGTCAAGGG
mHprt probe	FAM-ATCCAACAAAGTCTGGCCTGTATCCAAC-TAMRA
mGapdh forward	GAGATGATGACCCTTTTGGC
mGapdh reverse	GTCGTGGAGTCTACTGGTGTCTT

Supplementary Table 3. Primers used for ChIP assay.

Name	Sequence (5'-3')
hNOTCH3 ARE forward #	GGTTGGGCAGAGGAATAACA
hNOTCH3 ARE reverse #	GGCATGACGCAGGACTAGAT
hNOTCH3 enhancer forward ##	GTCTTTGCCTGGGTGCTTC
hNOTCH3 enhancer reverse ##	ACTGGCTGTGAGTTCCTGCT
hNOTCH3 ARE CRISPR forward ###	CCCTCAGTCCTGCATTTGG
hNOTCH3 ARE CRISPR reverse ###	GGGTTTGACATGGTGTGAC
hGCLM ARE forward	GGAGAGCTGATTCCAAACTG
hGCLM ARE reverse	GAGTAACGGTTACGAAGCAC
hGAPDH enhancer forward	CTCTGCTCTGGGTGGTCATT
hGAPDH enhancer reverse	CCTTTCTGGGATTGCCTTTC
hGATA1 exon3 forward	GCCTCAACTGTGTGTCCCAC
hGATA1 exon3 reverse	GAAGGTACTGGAAAAGTCAG
hFAM20B enhancer forward	CAAGCCCTATTGTGAACTGC
hFAM20B enhancer reverse	GACTGGGTGGGAATGGTTT
hZC3H12A enhancer forward	CCTCCACTGACCTCCAAAAC
hZC3H12A enhancer reverse	GGACCCAACTGAAAGCACTC
hC5AR1 enhancer forward	AGCCGTTTGCGTCTGAAG
hC5AR1 enhancer reverse	CAGTCCTTTCCTCTGGTTGA
hHGD enhancer forward	GGAAGGAAGGCTGGAGTACA
hHGD enhancer reverse	ACCCTCTTCTAAGGCCACCA
hTMOD3 enhancer forward	CAGATGGACGGCTTGTTACT
hTMOD3 enhancer reverse	ATGGAGAAATGCTGGAGGAA
RP11-383H13.1 enhancer forward	GCTTTTACCCTGGCTTTCAT
RP11-383H13.1 enhancer reverse	CGATGGTGGTGGGAATAC
hHMOX-1 enhancer forward	GGTAGGCAGGAGGAAGTGAA
hHMOX-1 enhancer reverse	GGGCAGATTGAGGTGGACT
hAKR1C1 enhancer forward	TTGTGTGTCAGCAGCCTCTC
hAKR1C1 enhancer reverse	TACAGGGCGAAGGAAAGCA
hAKR1C2 enhancer forward	TGTTGAAGCAAGTGACTGCC
hAKR1C2 enhancer reverse	ACATGAAAGCAAATGGGCCT

hNOTCH3 ARE primer set was used for NRF2 ChIP assay in A549, H460, H2023 cells.
hNOTCH3 enhancer primer set was used for H3K27ac ChIP assay in NSCLC cells except for NOTCH3 enhancer-disrupted H460 cells.
hNOTCH3 ARE CRISPR primer set was used for NRF2 and H3K27ac ChIP assay in *NOTCH3* enhancer-disrupted H460 cells, CEBPB and FOSL2 ChIP assay in *NRF2*-knockdown A549 cells, CEBPB ChIP assay in *NOTCH3* enhancer-disrupted A549 cells, NRF2 ChIP assay in *CEBPB*- and *FOSL2*-knockdown A549 cells and DEM induced H23 and H4006 cells.

Supplementary Table 4. Oligo DNAs for construction of gRNA expression vectors to disrupt *NOTCH3* AREs.

Name	Sequence (5’-3’)
hNOTCH3 gRNA1 forward	CACCGTGCTGAGTCAAGAGGCCCAT
hNOTCH3 gRNA1 reverse	AAACATGGGCCTCTTGACTCAGCAC
hNOTCH3 gRNA2 forward	CACCGAAGGGGCACACACTGACTCA
hNOTCH3 gRNA2 reverse	AAACTGAGTCAGTGTGTGCCCCTTC

Supplementary Table 5. Primer set for the PCR amplification of the DNA fragment spanning the gRNA target site.

Name	Sequence (5’-3’)
hNOTCH3 PCR1 forward #	GGTTGGGCAGAGGAATAACA
hNOTCH3 PCR1 reverse #	GGAGGGTTAGGAAGGAGAGG
hNOTCH3 PCR2 forward ##	TGAACCTGGGTCTCAGTGAC
hNOTCH3 PCR2 reverse ##	GGAGGGTTAGGAAGGAGAGG

hNOTCH3 PCR1 primer set was used for H460 and A549 cells.
hNOTCH3 PCR2 primer set was used for H2023.
