# Supplementary material for: Enhancer remodeling promotes tumor-initiating activity in NRF2-activated non-small cell lung cancers
Source: Nat Commun. 2020 Nov 20;11:5911. doi: 10.1038/s41467-020-19593-0 (PMC7679411; doi:10.1038/s41467-020-19593-0)
Supplement: Supplementary file 3 — Reporting Summary [file 41467_2020_19593_MOESM3_ESM.pdf]

nature research

Corresponding author(s): Hozumi Motohashi

Last updated by author(s): Sep 27, 2020

Reporting Summary

Nature Research wishes to improve the reproducibility of the work that we publish. This form provides structure for consistency and transparency in reporting. For further information on Nature Research policies, see our Editorial Policies and the Editorial Policy Checklist.

Statistics

For all statistical analyses, confirm that the following items are present in the figure legend, table legend, main text, or Methods section.

n/a	Confirmed	
☐	☒	The exact sample size (*n*) for each experimental group/condition, given as a discrete number and unit of measurement
☐	☒	A statement on whether measurements were taken from distinct samples or whether the same sample was measured repeatedly
☐	☒	The statistical test(s) used AND whether they are one- or two-sided<br>*Only common tests should be described solely by name; describe more complex techniques in the Methods section.*
☒	☐	A description of all covariates tested
☐	☒	A description of any assumptions or corrections, such as tests of normality and adjustment for multiple comparisons
☐	☒	A full description of the statistical parameters including central tendency (e.g. means) or other basic estimates (e.g. regression coefficient) AND variation (e.g. standard deviation) or associated estimates of uncertainty (e.g. confidence intervals)
☐	☒	For null hypothesis testing, the test statistic (e.g. *F*, *t*, *r*) with confidence intervals, effect sizes, degrees of freedom and *P* value noted<br>*Give P values as exact values whenever suitable.*
☒	☐	For Bayesian analysis, information on the choice of priors and Markov chain Monte Carlo settings
☒	☐	For hierarchical and complex designs, identification of the appropriate level for tests and full reporting of outcomes
☐	☒	Estimates of effect sizes (e.g. Cohen's *d*, Pearson's *r*), indicating how they were calculated

Our web collection on statistics for biologists contains articles on many of the points above.

Software and code

Policy information about availability of computer code

Data collection	TopHat Alignment (ver. 1.0.0) (https://jp.illumina.com/products/by-type/informatics-products/basespace-sequence-hub/apps/tophat-alignment.html)<br>Cufflinks software (ver. 2.3.1) (https://github.com/cole-trapnell-lab/cufflinks)<br>Bowtie2 (ver. 2.2.6) (http://bowtie-bio.sourceforge.net/bowtie2/manual.shtml)<br>SAMTools (ver. 1.3.1) (http://samtools.sourceforge.net/)<br>MACS2 (ver. 2.1.0.20151222) (https://github.com/macs3-project/MACS)
Data analysis	Microsoft Excel (ver. 16.41), Prism 7, JMP Pro 13, StatView 5.0J, ImageJ (ver. 1.45s),<br>GenometriCorr package (ver. 1.1.23) (http://genometricorr.sourceforge.net/)<br>BEDtools version v2.27.1 (ver. 2.27.1) (https://bedtools.readthedocs.io/en/latest/)<br>KentUtils (ver. 302) (https://github.com/ENCODE-DCC/kentUtils)<br>deepTools (ver. 3.0.1) (https://deeptools.readthedocs.io/en/develop/)<br>WiggleTools (ver. 1.2) (https://github.com/Ensembl/WiggleTools)

For manuscripts utilizing custom algorithms or software that are central to the research but not yet described in published literature, software must be made available to editors and reviewers. We strongly encourage code deposition in a community repository (e.g. GitHub). See the Nature Research guidelines for submitting code & software for further information.

Data

Policy information about availability of data

All manuscripts must include a data availability statement. This statement should provide the following information, where applicable:

- Accession codes, unique identifiers, or web links for publicly available datasets
- A list of figures that have associated raw data
- A description of any restrictions on data availability

RNA-seq data generated in this study have been deposited in GEO under the accession code GSE118841 (https://www.ncbi.nlm.nih.gov/geo/query/acc.cgi?acc=GSE118841) and GSE118842 (https://www.ncbi.nlm.nih.gov/geo/query/acc.cgi?acc=GSE118842). ChIP-seq data generated in this study have been deposited in GEO under the accession code GSE118840 (https://www.ncbi.nlm.nih.gov/geo/query/acc.cgi?acc=GSE118840). RNA-seq data and mutation data of lung adenocarcinoma samples from TCGA, PanCancer Atlas are available at https://www.cbioportal.org/study/summary?id=luad_tcga_pan_can_atlas_2018. RNA-seq data of lung squamous cell carcinoma samples from TCGA, PanCancer Atlas are available at https://www.cbioportal.org/study/summary?id=lusc_tcga_pan_can_atlas_2018. ChIP-seq data of transcription factors in A549 cells from ENCODE are available at https://www.encodeproject.org/search/?searchTerm=A549&type=Experiment&assay_title=TF+ChIP-seq&limit=all. The source data underlying Figs. 1a-c, g, 2a, c-e, 3b-g, 4a-f, 5c-e, 6f, 7b, d-f, 8c-f, h, 9a-f, Supplementary Figs. 1b, c, 2b, c, 3a, b, 5b, d, e, 6c, d, 7a-c, 10a-f, 11a-e, 12a-c, and 13c are provided as a Source Data File. All the other data supporting the findings of this study are available within the article and its supplementary information files and from the corresponding author upon reasonable request. A reporting summary for this article is available as a Supplementary Information file.

Field-specific reporting

Please select the one below that is the best fit for your research. If you are not sure, read the appropriate sections before making your selection.

☒ Life sciences ☐ Behavioural & social sciences ☐ Ecological, evolutionary & environmental sciences

For a reference copy of the document with all sections, see nature.com/documents/nr-reporting-summary-flat.pdf

Life sciences study design

All studies must disclose on these points even when the disclosure is negative.

Sample size	No sample size calculation was performed. Sample sizes (3-5 samples for cell line analyses, 8-12 samples for mouse experiment, 20-40 samples for human samples) were chosen based on previous experience and on what is common practice in the field.
Data exclusions	For immunoblot analysis of human tumor samples, three samples with very low protein recovery judged from tubulin band intensities were excluded from the analysis. This exclusion criteria is not a pre-established criteria. As a result, 17 samples were used for the data.
Replication	All experiments were independently repeated as described in the legends and methods and were reliably reproduced.
Randomization	For xenograft experiments, the recipient mice were randomly divided into test and control groups. Randomization was not relevant to other experiments because they were in vitro experiments.
Blinding	Histological evaluation of human tumor samples were blindly performed. For other experiments, blinding was not conducted during experiments because each experiment was performed by a single investigator and because collected data are quantitative and not influenced by investigator's bias.

Reporting for specific materials, systems and methods

We require information from authors about some types of materials, experimental systems and methods used in many studies. Here, indicate whether each material, system or method listed is relevant to your study. If you are not sure if a list item applies to your research, read the appropriate section before selecting a response.

Materials & experimental systems

n/a	Involved in the study	
☐	☒	Antibodies
☐	☒	Eukaryotic cell lines
☒	☐	Palaeontology and archaeology
☐	☒	Animals and other organisms
☐	☒	Human research participants
☒	☐	Clinical data
☒	☐	Dual use research of concern

Methods

n/a	Involved in the study	
☐	☒	ChIP-seq
☒	☐	Flow cytometry
☒	☐	MRI-based neuroimaging

Antibodies

Antibodies used	ChIP assay;<br>anti-H3K27ac antibody (MABI0309, MAB Institute),<br>anti-NRF2 antibody (#12721, Cell Signaling Technology),<br>anti-FOSL2 antibody (#19967S, Cell Signaling Technology),<br>anti-CEBPB antibody (sc-150 X, Santa Cruz)<br>rabbit IgG (#55944, Cappel/SKU 0855944, MP Biomedicals)<br>Immunoblot analysis;<br>anti-NRF2 (sc-13032X, Santa Cruz),<br>anti-NOTCH3 (ab23426, Abcam),<br>anti-CEBPB antibody (sc-150 X, Santa Cruz),<br>anti-Tubulin (T9026, Sigma),<br>anti-Lamin B (sc-6217, Santa Cruz),<br>anti-FOSL2 antibody (#19967S, Cell Signaling Technology)<br>anti-KEAP1 antibody (#111, Watai et al., 2007; now commercially available as MABS514, Merck)<br>Histological analysis;<br>NOTCH3 (ab60087, Abcam),<br>NRF2 (sc-13032X, Santa Cruz),<br>CEBPB (SAB4500112, Sigma-Aldrich)<br>Mouse cell removal for serial transplantation;<br>CD31 (#13-0311-82, eBioscience),<br>CD45 (#13-0451-85, eBioscience),<br>MCH class I antibody (ab95572, Abcam)<br>Dilution and amount of antibodies have been described in the Methods section.
Validation	https://www.activemotif.com/catalog/details/39685/histone-h3-acetyl-lys27-antibody-mab-clone-mabi-0309<br>https://media.cellsignal.com/pdf/12721.pdf<br>https://media.cellsignal.com/pdf/19967.pdf<br>https://www.citeab.com/antibodies/781587-sc-150-c-ebp-antibody-c-19<br>https://www.citeab.com/antibodies/819972-sc-13032-nrf2-antibody-h-300<br>https://www.abcam.com/notch3-antibody-ab23426.html<br>https://www.sigmaaldrich.com/catalog/product/sigma/t9026?lang=en&region=US<br>http://datasheets.scbt.com/sc-6217.pdf<br>https://www.abcam.com/notch3-antibody-ab60087.html<br>https://www.sigmaaldrich.com/catalog/product/sigma/sab4500112?lang=en&region=US<br>https://www.thermofisher.com/antibody/product/CD31-PECAM-1-Antibody-clone-390-Monoclonal/13-0311-82<br>https://www.thermofisher.com/antibody/product/CD45-Monoclonal-Antibody-30-F11-Biotin-eBioscience/13-0451-85<br>https://www.abcam.co.jp/mhc-class-i-antibody-34-1-2s-fitc-ab95572.html<br>https://www.merckmillipore.com/JP/en/product/Anti-Keap1-Antibody-clone-144,MM_NF-MABS514<br>https://www.mpbio.com/us/purified-rabbit-igg-50-mg

Eukaryotic cell lines

Policy information about cell lines

Cell line source(s)	A549, H460, H2023, H1944, H1650, HCC4006, H23;ATCC<br>ABC1; JCRB<br>CORL105; Sigma-Aldrich<br>293FT; Invitrogen
Authentication	None of the cell lines used were authenticated.<br>In our study, all cell lines were divided into two categories, NRF2 is constitutively accumulated or not, which was all consistent with their cell line names and their KEAP1 gene mutation statuses.
Mycoplasma contamination	All cell lines were negative for mycoplasma infection.
Commonly misidentified lines<br>(See ICLAC register)	No commonly misidentified cell lines were used in the study.

Animals and other organisms

Policy information about studies involving animals; ARRIVE guidelines recommended for reporting animal research

Laboratory animals	Four-week-old male BALB/cAJcl-nu/nu mice<br>8-12 week-old male Keap1-knockdown (Okawa et al., 2006) and their control wild-type mice in C57BL/6 background
Wild animals	No wild animals were used in the study.
Field-collected samples	No field collected samples were used in the study.

Ethics oversight	The Standards for Human Care and Use of Laboratory Animals of Tohoku University<br>Guidelines for Proper Conduct of Animal Experiments by the Ministry of Education, Culture, Sports, Science, and Technology of Japan

Note that full information on the approval of the study protocol must also be provided in the manuscript.

Human research participants

Policy information about studies involving human research participants

Population characteristics	Tumor tissue specimens for histological analysis were obtained from 41 lung adenocarcinoma patients who underwent surgical resection without preoperative treatments, irradiation or chemotherapy, between 2003 and 2004 in the Department of Thoracic Surgery at Tohoku University Hospital.
Recruitment	The 41 lung adenocarcinoma patients were randomly selected.
Ethics oversight	Ethics Committees at Tohoku University Graduate School of Medicine

Note that full information on the approval of the study protocol must also be provided in the manuscript.

ChIP-seq

Data deposition

☒ Confirm that both raw and final processed data have been deposited in a public database such as GEO.

☒ Confirm that you have deposited or provided access to graph files (e.g. BED files) for the called peaks.

Data access links<br>*May remain private before publication.*	GEO (GSE118840)<br>https://www.ncbi.nlm.nih.gov/geo/query/acc.cgi?acc=GSE118840
Files in database submission	GSM3349035_000062Y_peaks.broadPeak.gz<br>GSM3349036_000068Y_peaks.broadPeak.gz<br>GSM3349037_000295Y_peaks.broadPeak.gz<br>GSM3349038_000296Y_peaks.broadPeak.gz<br>GSM3349039_000299Y_peaks.broadPeak.gz<br>GSM3349040_000300Y_peaks.broadPeak.gz<br>GSE118840_NRF2_siRNA.bw<br>GSE118840_control_siRNA.bw
Genome browser session<br>(e.g. UCSC)	No longer applicable

Methodology

Replicates	Three biological replicates were obtained, which showed good agreement.
Sequencing depth	101-base single-end reads for all.<br>000062Y (A549-ctrl_1) total reads:20,692,275 mapper reads:18,456,128<br>000068Y (A549-KD_1) total reads:21,984,756 mapper reads:19,275,035<br>000295Y (A549-ctrl_2) total reads:43,462,912 mapper reads:39,291,456<br>000296Y (A549-KD_2) total reads:45,474,163 mapper reads:40,341,743<br>000299Y (A549-ctrl_3) total reads:47,032,764 mapper reads:43,029,764<br>000300Y (A549-KD_3) total reads:45,770,026 mapper reads:41,185,858<br>000307Y (A549-input_1) total reads:45,427,495 mapper reads:39,930,730<br>000308Y (A549-input_2) total reads:46,457,739 mapper reads:40,957,700
Antibodies	anti-H3K27ac antibody (MABI0309, MAB Institute)<br>https://www.activemotif.com/catalog/details/39685/histone-h3-acetyl-lys27-antibody-mab-clone-mabi-0309
Peak calling parameters	Read mapping and peak calling were conducted by using Bowtie2 version 2.2.6 and MACS2 version 2.1.0.20151222 with default parameters, respectively.
Data quality	000062Y (A549-ctrl_1) total peaks:27,941 peaks at FDR 5% and above 5-fold enrichment:9,005<br>000068Y (A549-KD_1) total peaks:23,885 peaks at FDR 5% and above 5-fold enrichment:5,836<br>000295Y (A549-ctrl_2) total peaks:34,747 peaks at FDR 5% and above 5-fold enrichment:11,402<br>000296Y (A549-KD_2) total peaks:34,986 peaks at FDR 5% and above 5-fold enrichment:12,678<br>000299Y (A549-ctrl_3) total peaks:41,571 peaks at FDR 5% and above 5-fold enrichment:16,340<br>000300Y (A549-KD_3) total peaks:39,108 peaks at FDR 5% and above 5-fold enrichment:13,796
Software	Reads with MAPQ <20 were removed using SAMTools version 1.3.1. The ENCODE blacklist was applied to all obtained peaks to filter out possible non-functional signals. ChIP-seq peak visualization was performed using the Integrative Genomic Viewer. The bedGraph files were converted into BigWig files by using KentUtils version 302, then deepTools version 3.0.1 was adopted to draw aggregation plots and heat maps. We used BEDtools version v2.27.1 to identify overlaps.
